# Association genetics of phenolic needle compounds in Norway spruce with variable susceptibility to needle bladder rust

**DOI:** 10.1007/s11103-017-0589-5

**Published:** 2017-02-11

**Authors:** Andrea Ganthaler, Wolfgang Stöggl, Stefan Mayr, Ilse Kranner, Silvio Schüler, Elisabeth Wischnitzki, Eva Maria Sehr, Silvia Fluch, Carlos Trujillo-Moya

**Affiliations:** 1 Institute of Botany, University of Innsbruck, Sternwartestrasse 15, 6020 Innsbruck, Austria; 2 alpS – Centre for Climate Change Adaptation, Grabenweg 68, 6020 Innsbruck, Austria; 3 Department of Forest Genetics, Federal Research and Training Centre for Forests, Natural Hazards and Landscapes (BFW), Seckendorff-Gudent-Weg 8, 1131 Vienna, Austria; 4 Health and Environment Department, AIT Austrian Institute of Technology GmbH, Konrad-Lorenz-Strasse 24, 3430 Tulln, Austria

**Keywords:** Association genetics, Pathogen defence, Phenolic metabolites, *Picea abies*, Resistance, Single nucleotide polymorphisms

## Abstract

**Key message:**

Accumulation of phenolic needle metabolites in Norway spruce is regulated by many genes with small and additive effects and is correlated with the susceptibility against fungal attack.

**Abstract:**

Norway spruce accumulates high foliar concentrations of secondary phenolic metabolites, with important functions for pathogen defence responses. However, the molecular genetic basis underlying the quantitative variation of phenolic compounds and their role in enhanced resistance of spruce to infection by needle bladder rust are unknown. To address these questions, a set of 1035 genome-wide single nucleotide polymorphisms (SNPs) was associated to the quantitative variation of four simple phenylpropanoids, eight stilbenes, nine flavonoids, six related arithmetic parameters and the susceptibility to infection by *Chrysomyxa rhododendri* in an unstructured natural population of Norway spruce. Thirty-one significant genetic associations for the flavonoids gallocatechin, kaempferol 3-glucoside and quercetin 3-glucoside and the stilbenes resveratrol, piceatannol, astringin and isorhapontin were discovered, explaining 22–59% of phenotypic variation, and indicating a regulation of phenolic accumulation by many genes with small and additive effects. The phenolics profile differed between trees with high and low susceptibility to the fungus, underlining the importance of phenolic compounds in the defence mechanisms of Norway spruce to *C. rhododendri*. Results highlight the utility of association studies in non-model tree species and may enable marker-assisted selection of Norway spruce adapted to severe pathogen attack.

**Electronic supplementary material:**

The online version of this article (doi:10.1007/s11103-017-0589-5) contains supplementary material, which is available to authorized users.

## Introduction

The chemical composition of tissues plays important roles in the defence of plants against herbivory and pathogens. Of the defence-related plant secondary metabolites, phenolic compounds are particularly important for quantitative resistance to fungal pathogens, which are the most important group of tree pathogens (Hammerschmidt 2005; Witzell and Martín 2008). Phenolic compounds comprise a structurally and functionally diverse group of metabolites characterised by aromatic hydrocarbon ring(s) and usually at least one attached hydroxyl group. Phenolics can have fungicide or antioxidant properties, can be involved in resistance mechanisms as precursors of defence-related compounds or polymers, and can modulate the activity of other phytochemicals (Schultz and Nicolas 2000; Treutter 2006). In addition, they can be incorporated into the cell wall and form mechanical barriers (Cvikrová et al. 2008). Phenolic metabolites are considered an important part of both constitutive as well as inducible defence mechanisms (Chong et al. 2009).

In the Pinaceae family, much attention has been given to phenylpropanoid metabolism (Bernards and Båstrup-Spohr 2008; Danielsson et al. 2011), which provides precursors of compounds involved in resistance to pathogens, such as stilbenes, flavonoids and lignin (Chong et al. 2009; Jeandet et al. 2010; Fossdal et al. 2012). In Norway spruce (*Picea abies* (L.) H.Karst), several phenolic compounds were found to be associated with resistance to insects, herbivory and pathogen attack, or showed increased concentration following infection and wounding (Lindberg et al. 1992; Brignolas et al. 1995; Bahnweg et al. 2000; Evensen et al. 2000; Delvas et al. 2011; Hammerbacher et al. 2011, 2013; Fossdal et al. 2012; Schiebe et al. 2012; Krajnc et al. 2014). Furthermore, attempts have been made to use phenolic metabolites as predictors of spruce resistance to insect and microbial attack (Lieutier et al. 1997, 2003; Brignolas et al. 1998). Nevertheless, some clones with high levels of stilbenes have been described as susceptible to *Endoconidiophora polonica* (Hammerbacher et al. 2013).

The fungus *Chrysomyxa rhododendri* (DC.) de Bary (De Bary 1879; Gäumann 1959; Ganthaler et al. 2014) is a frequent and serious pathogen affecting trees in large areas of the European Alps. The rust fungus undergoes a host shift between rhododendrons and Norway spruce and infects the current-year needles in the first weeks after flushing, causing an intensive yellow discoloration after 3–4 weeks and defoliation at the end of summer. In the past decade, following a long stable period, infection intensity and affected forest areas increased significantly (Ganthaler et al. 2014), and are thought to be promoted by the expansion of the telial host rhododendron, and by global warming and more favourable conditions for the pathogen (Ganthaler and Mayr 2015). In the investigation area Tyrol, more than 20,000 ha of spruce forest were repeatedly infected since 2009 (Fuchs et al. 2016). Infections cause anatomical, morphological and physiological modifications of attacked trees, leading to reduced timber yield and notable problems with natural regeneration and afforestation (Ganthaler et al. 2014). Dry mass accumulation in 3-year-old seedlings, for example, was reduced by 58% when infected in two consecutive years (Plattner et al. 1999). These problems are serious, as Norway spruce is a widespread and socio-economically and ecologically important tree species in European subalpine forests, with important protective function. However, high variation of susceptibility of Norway spruce to *C. rhododendri* infection was repeatedly reported (Dufrénoy 1932; Oechslin 1933; Mayr et al. 2010) and interestingly, even in years with severe outbreaks individual trees with distinctly lower degree of infection than the surrounding trees were observed, suggesting that they have enhanced pathogen resistance. Importantly, this lower susceptibility clearly benefits the trees, which have higher net photosynthesis, lower cuticular conductance, and higher growth rates compared to highly infected trees (Mayr et al. 2001, 2010). The underlying resistance mechanisms, including metabolic background and genetic control, are not understood, but there is evidence that phenolic secondary compounds may limit the growth of rust fungi immediately after infection and prevent the development of infection symptoms (Hakulinen et al. 1999; Hjältén et al. 2007). Besides a direct fungicidal effect, phenolics may be incorporated in the plant cell wall and influence the interaction with the biotrophic fungus (Matern and Kneusel 1988).

Variation of plant secondary metabolites depends upon both, genetic and environmental factors (Hamilton et al. 2001; Andrew et al. 2007; Külheim et al. 2011). However, several investigations have demonstrated considerable heritability for constitutive and induced phenolic concentrations (Witzell and Martín 2008) and suggested a regulation by allelic variants of multiple genes, but few studies have addressed the molecular basis of quantitative differences (Keeling et al. 2008; Chan et al. 2010). Association mapping is a powerful tool to identify marker-trait associations (MTAs) in model as well as non-model tree species (Neale and Savolainen 2004; Budde et al. 2014). Natural populations adapted to extreme environments, like the alpine timberline, are ideal for the identification of ecologically relevant genetic variation. In addition, coniferous forest trees exhibit several important advantages such as showing high levels of genetic diversity, random mating and large populations, which lead to low inbreeding, highly efficient gene flow, low population structure and rapid decay of linkage disequilibrium (Neale and Savolainen 2004). In coniferous forest trees, associations have been reported for several phenotypic traits, including wood properties (González-Martínez et al. 2007; Dillon et al. 2010; Beaulieu et al. 2011; Westbrook et al. 2013), growth and wood chemistry (Lepoittevin et al. 2012), serotiny (Budde et al. 2014), carbon isotope discrimination (González-Martínez et al. 2008; Cumbie et al. 2011), cold hardiness and bud set timing (Eckert et al. 2009; Holliday et al. 2010), but rarely for cellular phenotypes such as metabolite concentrations (Eckert et al. 2012) or disease resistance (Quesada et al. 2010). For Norway spruce, only studies focusing on chlorophyll fluorescence, frost resistance, height, diameter, bud burst (Romsakova et al. 2012) and bud set (Chen et al. 2012a) are available. However, the genetic basis of variation in metabolite concentrations and its relation to foliar pest infections is largely unknown.

The present study was based on an initiative of forest authorities to identify trees with distinct lower degree of *C. rhododendri* infection and should provide deeper insights into the relationship between genetic background, phenolic composition and infection. Therefore, a genome-wide SNP array set with 3257 polymorphic SNPs was used to conduct association analysis of Norway spruce phenolic needle compounds and susceptibility to needle bladder rust in an unstructured population of 63 trees from different provenances in Tyrol, Austria. To the best of our knowledge, this is the first study reporting genetic markers for quantitative variation of stilbenes and flavonoids in Norway spruce. This work may help identifying favourable alleles for marker-assisted selection of Norway spruce adapted to severe pathogen attack.

## Materials and methods

### Association population and sampling

Sixty-three Norway spruce trees from different locations in Tyrol, Austria were used (Supplemental Table S1). The individuals were identified by the Tyrolean Forest Department in cooperation with the Institute of Botany of the University of Innsbruck in order to have an association population with a high variation in the susceptibility to *C. rhododendri*, ranging from heavily damaged to nearly unaffected trees. Number of trees was limited by the complex logistic planning of contemporaneous sampling in impassable subalpine terrain. Trees were between 25 and 120 years old and all were located next to the alpine timberline between 1401 and 1814 m above sea level. From each of these trees, ten twigs of the five uppermost whorls were harvested in April 2013 and transported to the laboratory. The upper crowns were not shielded by neighbour trees from airborne spores and thus observed infection degree was expected to reflect the trees’ susceptibility. Previous-year fully developed and healthy needles were cut randomly from the twigs and stored immediately at 80 °C for metabolic and genetic analyses.

### Assessment of the infection degree

*Chrysomyxa rhododendri* infection degrees for all trees were determined by assessing the percentage of needle loss due to infection on sampled twigs on a scale of 1–5 (1: 0–20%, 2: 21–40%, 3: 41–60%, 4: 61–80%, 5: 81–100% needle loss) for the 4 years 2009–2012 (compare Oberhuber et al. 1999). Mean infection values for the individual trees were calculated and used for association analysis. In addition, the binary trait low/high susceptibility was applied: trees with at least 20% lower infection degree over the four analysed years compared to the immediately surrounding trees in the forest stand were defined as showing ‘low susceptibility’, trees with continuous high infection intensities representative for the infection degree in the study area as showing ‘high susceptibility’ (compare Table S1). Relative assessment of susceptibility in the forest stand and monitoring of infection degrees over several years in this context is important, as the percentage of infected needles is influenced by local spore densities and weather conditions during the infection period (Ganthaler and Mayr 2015). From each forest district involved in the study, at least one tree with high and one with low susceptibility were included.

### Identification and quantification of phenolic compounds

Healthy 1-year-old needles of the year 2012 were freeze-dried for 72 h and homogenized for 8 min at 2000 rpm in a microdismembrator (Mikro-Dismembrator U, Braun Biotech International, Melsungen, Germany) using 7 ml Teflon grinding capsules and one agate ball of 1 cm diameter. To avoid warming of the sample, capsules containing the needles were submerged in liquid nitrogen for 2 min before grinding, and to avoid sublimation of water on the powder, the capsules were allowed to equilibrate with room temperature in a desiccator over silica gel before opening (Bailly and Kranner 2011). The powder was transferred into Eppendorf vials and 10 mg was extracted two times for 20 min each at 50 °C on a thermo mixer with 600 rpm, with 1 ml 95% (v/v) ethanol, containing 2 µMol l^−1^ orientin, pinosylvin and naringin as internal standards for quantification, followed by a centrifugation for 10 min at 12,000×*g*. The supernatants were merged and diluted 1:2 and 1:50 with ethanol and water to obtain a 50:50 ethanol/water (v/v %) extract and to consider the different concentration ranges of the metabolites in the extract. Twenty-one phenolic compounds (Table 1) were identified and quantified by liquid chromatography-mass spectrometry (LC-MS), using an ekspert ultraLC 100 UHPLC system coupled with a QTRAP 4500 mass spectrometer (both from AB SCIEX, Framingham, MA, USA). Individual metabolites were detected and quantified using calibration curves of authentic standards. For compounds separation, a reversed-phase UHPLC column (NUCLEODUR C18 Pyramid, EC 50/2, 50 × 2 mm, 1.8 µm, Macherey–Nagel, Düren, Germany) with a 4 × 2 mm guard column was used. Run time was set to 8 min and mobile phases were 0.1% formic acid (v/v) (A) and acetonitrile (B), starting with 5% B followed by a gradient to 70% B (5 min), rinsing at 100% B (5:01 to 6 min) and equilibration at 5% B (6:30 to 8 min). The injection volume was set to 1 µl, the flow rate to 0.5 ml min^−1^ and column temperature to 30 °C. Compounds were detected by the mass spectrometer operated in negative ion mode using multiple reaction monitoring (MRM; Supplemental Table S2). Ion spray voltage was set to 4.5 kV, gas 1–40 psi and gas 2–50 psi at a temperature of 500 °C. Both quadrupole mass analysers were operated at unit resolution. Peaks were automatically detected based on retention time and MRM transition. Peak areas were normalized relative to the internal standards to account for variations during sample preparation and analysis, and concentration was calculated according to the compound-specific calibration curves established with authentic standards using the software Analyst and MultiQuant (AB SCIEX, Framingham, MA, USA).

**Table 1:** Analysed phenolic compounds and internal standards

Compound	Class	MW	Formula
*cis-*/*trans*-Astringin	Stilbene	406.38	C_20_H_22_O_9_
Catechin	Flavonoid	290.27	C_15_H_14_O_6_
Chlorogenic acid	Phenylpropanoid	354.31	C_16_H_18_O_9_
Gallic acid	Phenylpropanoid	170.12	C_7_H_6_O_5_
Gallocatechin	Flavonoid	306.27	C_15_H_14_O_7_
*cis-*/*trans*-Isorhapontin	Silbene	420.41	C_21_H_24_O_9_
Kaempferol	Flavonoid	286.23	C_15_H_10_O_6_
Kaempferol 3-glucoside	Flavonoid	448.38	C_21_H_20_O_11_
Naringenin	Flavonoid	272.26	C_15_H_12_O_5_
*trans*-Piceatannol	Stilbene	244.24	C_14_H_12_O_4_
*cis-*/*trans*-Piceid	Stilbene	390.38	C_20_H_22_O_8_
Picein	Phenylpropanoid	298.29	C_14_H_18_O_7_
Quercetin	Flavonoid	302.24	C_15_H_10_O_7_
Quercetin 3-glucoside	Flavonoid	464.38	C_21_H_20_O_12_
Quercitrin	Flavonoid	448.38	C_21_H_20_O_11_
*trans-*Resveratrol	Stilbene	228.24	C_14_H_12_O_3_
Shikimic acid	Cyclohexene	174.15	C_7_H_10_O_5_
Taxifolin	Flavonoid	304.25	C_15_H_12_O_7_
Internal standards			
Naringin	Flavonoid	580.53	C_27_H_32_O_14_
Orientin	Flavonoid	448.38	C_21_H_20_O_11_
Pinosylvin	Stilbene	212.24	C_14_H_12_O_2_

Compound name, compound classes, molecular weight (MW) and chemical formula (formula) are given

Correlations were analysed using the Pearson (normally distributed data) or the Spearman Rank (not normally distributed data) correlation coefficient. Comparisons of the subgroups showing high and low susceptibility were performed, after testing normality with the Kolmogorov–Smirnov test, with *t* test (normally distributed data) or the Mann–Whitney *U* test (not normally distributed data). All tests were performed at a probability level of 5% using SPSS (version 21; SPSS, IL, USA). All values are given as mean ± SE.

### SNP microarray design

A custom Illumina InfiniumHD iSelect BeadChip comprising 3257 SNPs (assays) was developed by merging SNPs from a number of different resequencing and genotyping projects. The majority of SNPs (1742) were originally identified in and designed for *Picea glauca* and later also tested on and found to be variable in a small number of *P. abies* individuals (Pavy et al. 2013). Additional 583 SNPs came from a mRNA sequencing of a single individual using Illumina technology (Chen et al. 2012b), and 311 SNPs were chosen from an mRNA sequencing approach (Heer et al. 2016). Further 228 SNPs stemmed from the sequencing of pooled PCR products using Illumina next-generation sequencing technology (Chen et al. 2016), 178 SNPs originally identified in *Picea glauca* were tested on *P. abies* using Illumina’s Golden Gate technology (Chen et al. 2012a), and 141 SNPs came from Sanger resequencing efforts, which had been sequenced and analysed in Heuertz et al. (2006), Chen et al. (2010), and Källman et al. (2014). In addition, 57 SNPs derived from the CRSP project headed by David Neale (http://dendrome.ucdavis.edu/NealeLab/crsp/), and 17 SNPs were designed based on loci available at Genbank from a population resequencing study of different conifer species (Guillet-Claude et al. 2004). A detailed list of the compiled assays is given in Supplemental Table S3.

### SNP genotyping

InfiniumBeadChips were manufactured by Illumina in a 24 × 1 format. For each sample, genomic DNA was extracted from freeze-dried needle tissue using a CTAB protocol (van der Beek et al. 1992) with minor modifications made for the processing of 96-well deep well plates. DNA concentrations were quantified on a 0.8% agarose gel. At the IMGM Laboratories GmbH, SNP genotyping was conducted according to the manufacturer’s recommendations and the microarray signals were detected on Illumina’s iScan System. All SNP data analyses were conducted using GenomeStudio v. 2011.1 (Illumina).

### Population structure and relatedness

Three hundred and fifty-six polymorphic neutral SNPs were used to investigate population stratification and relatedness between individuals as they can lead to false positive detection during association analysis. The selection of neutral SNPs followed a two-step procedure: first, only those SNPs located outside of genes or within gene introns were selected; second, these markers were filtered for a minimum call rate of 60 trees out of 63 and a minor allele frequency (MAF) above 0.2. Population stratification was first investigated with the Bayesian model-based software STRUCTURE (Falush et al. 2003) which is used to infer distinct populations and to assign individuals to the identified populations. The model allows admixture and correlated allele frequencies and was run with a burn-in period of 10^4^ and 50^5^ of Markov chain Monte Carlo replications after burn-in (run length). Ten independent runs (iterations) were conducted for each putative number of cluster K. Sampling location information was considered by applying the prior model parameter (LOCPRIOR) to the population model (Hubisz et al. 2009) and possible K’s tested ranged from 1 to 15 (number of locations). Alternative scenarios without LOCPRIOR were also tested. For each scenario, the Structure Harvester (Earl and vonHoldt 2012) was used to estimate the most probable number of K’s using Evanno’s method (Evanno et al. 2005). Population stratification was also studied by principal component analysis (PCA) using TASSEL (Bradbury et al. 2007), where the correlation matrix of genotype data was applied as a basis for analysis. In order to have a general overview on the relatedness between individuals showing high and low susceptibility, a cladogram was built up using neighbour joining implemented in TASSEL. Archaeopteryx plugin was used to draw the tree.

### Association test

Association analysis was performed using TASSEL (Bradbury et al. 2007). Markers with MAF less than 15% and more than 10% of missing data were excluded for the association test. A final set of 1035 SNP was selected to calculate MTAs using three models to evaluate the effects of population stratification: first, a model without correction (Generalized Linear Model: GLM), then models correcting for population stratification estimated by STRUCTURE (Q) and by PCA (P). Due to multiple testing, p value threshold was corrected with the standard Bonferroni procedure (*0.1 < p = 9.66 × 10^−5^; **0.05 < p = 4.83 × 10^−5^; ***0.01 < p = 9.66 × 10^−6^). The amount of variation explained by a SNP (Rsq_Marker) was calculated for each significant association using a simple general linear model. The q-value for each marker was calculated to adjust for the false discovery rate (Storey and Tibshirani 2003) using “qvalue” version 1.40.0 (Dabney, A. and Storey, J) with R (http://www.r-project.org/). Q-value threshold of 10% was used to declare significant associations.

Q-Q plots were used to assess the number and magnitude of observed associations between SNPs and traits under study, compared to the association statistics expected under the null hypothesis of no association. This procedure resulted in −log10 p values that were ranked in the order from smallest to largest on the y-axis and plotted against the distribution that would be expected under the null hypothesis of no association on the x-axis. Deviations from the identity line suggest either that the assumed distribution is incorrect (population structure not included in the model) or that the sample contains values arising due to other manner, as most likely by true associations (Burton et al. 2007; Pearson and Manolio 2008).

Finally, to create an overall composite measure for all chemical traits, a correlation matrix of the chemical data was used as a basis for a PCA performed in TASSEL. Numerical imputation by computing mean of respective traits was used to fill missing values. In order to explore the linkage disequilibrium (LD) among markers, we calculated the correlations between alleles at two SNP loci r_2_ within contigs using TASSEL.

## Results

### Variation in infection degree and phenolic needle metabolites

Within the association population, *C. rhododendri* infection degrees during the four observed years varied from Norway spruce trees with single infected needles to individuals with a needle loss of more than 65%. On average, trees classified as highly susceptible exhibited twice as high infections as trees with low susceptibility (Table 2). No correlation of the degree of infection with tree age or elevation was found (data not shown).

**Table 2:** Variation for the average *Chrysomyxa*-infection degree over the years 2009–2012 (classes 1–5) and concentration of analysed phenolic compounds (µMol g^−1^ dry weight), *cis*/*trans*-ratios and -sums

	Population	Min–max	Low susceptibility	High susceptibility
Infection degree	2.02 ± 0.11	1.00–3.75	**1.55** ± **0.06**	**3.12** ± **0.14**
*trans*-Astringin	31.69 ± 2.40	3.10–85.21	31.30 ± 2.98	32.59 ± 4.03
*cis*-Astringin	2.48 ± 0.24	0.11–7.88	2.25 ± 0.29	3.02 ± 0.40
Astringin ratio	8.00 ± 0.51	1.81–19.01	**7.33** ± **0.63**	**9.55** ± **0.75**
Astringin sum	34.17 ± 2.56	3.32–86.75	33.55 ± 3.18	35.62 ± 4.35
Catechin	30.08 ± 1.00	14.79–60.52	30.02 ± 1.02	30.24 ± 2.38
*trans*-Isorhapontin	4.65 ± 0.48	0.07–12.80	4.63 ± 0.57	4.71 ± 0.90
*cis*-Isorhapontin	1.08 ± 0.15	0.02–5.15	0.95 ± 0.15	1.37 ± 0.34
Isorhapontin ratio	19.91 ± 1.40	1.62–44.98	**18.01** ± **1.62**	**24.10** ± **2.53**
Isorhapontin sum	5.70 ± 0.61	0.07–17.80	5.53 ± 0.70	6.08 ± 1.22
Kaempferol 3-glucoside	1.09 ± 0.05	0.38–2.38	1.07 ± 0.06	1.12 ± 0.10
Picein	84.52 ± 6.60	16.02–240.05	81.54 ± 6.86	91.44 ± 15.31
Quercetin 3-glucoside	0.42 ± 0.03	0.17–1.59	0.43 ± 0.04	0.38 ± 0.04
Gallocatechin	14.62 ± 0.65	5.44 ± 26.72	**13.72** ± **0.67**	**16.70** ± **1.43**
Piceatannol	0.78 ± 0.16	0.01–5.63	0.66 ± 0.14	1.04 ± 0.40
*trans*-Piceid	0.79 ± 0.09	0.02–2.98	0.77 ± 0.12	0.86 ± 0.15
*cis*-Piceid	0.10 ± 0.01	0.00–0.50	**0.09** ± **0.02**	**0.13** ± **0.02**
Piceid ratio	12.47 ± 0.94	1.39–35.41	**11.35** ± **1.12**	**14.95** ± **1.64**
Piceid sum	0.89 ± 0.10	0.02–3.48	0.85 ± 0.13	0.99 ± 0.17
Shikimic acid	193.27 ± 5.70	30.54–299.60	193.86 ± 5.64	191.91 ± 13.98
*trans*-Resveratrol	0.06 ± 0.01	0.00–0.64	0.05 ± 0.01	0.10 ± 0.04

Given are mean ± SE and minimum and maximum values for the whole population and subgroups showing high and low susceptibility. Compounds with a concentration <0.1 µMol g^−1^ DW are not shown, significant differences between subgroups are shown in bold. For details see “Materials and methods” section

All analysed phenolic compounds were detected in the needle samples, but seven showed concentrations below the quantification limit of about 0.02 µMol g^1^ dry weight: chlorogenic acid, gallic acid, kaempferol, quercetin, quercitrin, naringenin and taxifolin. Consequently, these compounds were excluded from further analysis. The remaining eight stilbenes, four flavonoids and two simple phenylpropanoids showed large variation in concentration within the association population, apparent by the minimum and maximum values given in Table 2, and several trees showed extremely high levels of individual compounds. The most abundant metabolites were shikimic acid, picein, astringin and catechin (the complete phenotype dataset is given in Supplementary Table S7).

Needles from trees with lower susceptibility were characterised by significantly lower concentrations of gallocatechin (p = 0.036) and *cis*-piceid (p = 0.030), as well as lower *cis*/*trans*-ratios of the stilbenes astringin (p = 0.043), isorhapontin (p = 0.027) and piceid (p = 0.047) compared to highly susceptible trees (see Table 2). Accordingly, the infection degree was significantly correlated with the *cis*/*trans*-ratios of astringin (p = 0.003), isorhapontin (p = 0.004) and piceid (p = 0.026), but not with *cis*-piceid and gallocatechin (Table 3).

**Table 3:** Correlations between the concentrations of measured phenolic needle compounds, including related arithmetic parameters and infection degree

	Infection degree	*trans*-Astringin	*cis*-Astringin	Ratio astringin	Sum astringin	Catechin	*trans*-Isorhapontin	*cis*-Isorhapontin	Ratio isorhapontin	Sum isorhapontin	Kaempferol 3-glucoside	Picein	Quercetin 3-glucoside	Gallo-catechin	Picea-tannol	*trans*-Piceid	*cis*-Piceid	Ratio piceid	Sum piceid	Shikimic acid
*trans*-Astringin	−0.008																			
*cis*-Astringin	0.220	**0.663**																		
Ratio astringin	**0.371**	−0.067	**0.606**																	
Sum astringin	0.011	**0.998**	**0.714**	−0.005																
Catechin	−0.049	−0.044	0.026	0.055	−0.039															
*trans*-Isorhapontin	0.108	**0.411**	0.209	−0.128	**0.404**	−0.154														
*cis*-Isorhapontin	0.232	**0.400**	**0.477**	0.192	**0.396**	−0.143	**0.905**													
*Ratio* isorhapontin	**0.361**	0.131	**0.607**	**0.777**	0.179	−0.083	**0.348**	**0.712**												
Sum isorhapontin	0.128	**0.402**	**0.262**	−0.048	**0.401**	−0.147	**0.992**	**0.933**	**0.433**											
Kaempferol 3-glucoside	0.233	0.037	0.185	0.222	0.052	0.037	0.025	0.102	0.170	0.023										
Picein	0.220	0.160	**0.534**	**0.587**	0.200	0.158	0.058	0.196	**0.443**	0.104	0.207									
Quercetin 3-glucoside	0.036	0.095	−0.008	−0.094	0.096	**0.293**	−0.074	−0.163	−**0.256**	−0.094	**0.537**	0.066								
Gallocatechin	0.222	−0.137	0.008	0.147	−0.128	−**0.316**	−0.078	−0.004	0.054	−0.063	0.143	0.187	0.018							
Piceatannol	−0.092	**0.785**	**0.453**	−**0.350**	**0.759**	−0.160	**0.547**	**0.404**	0.014	**0.535**	−0.058	−0.236	0.005	−**0.315**						
*trans*-Piceid	0.054	**0.901**	**0.637**	−0.154	**0.891**	−0.117	**0.614**	**0.481**	0.135	**0.605**	0.076	0.056	0.070	−0.163	**0.807**					
*cis*-Piceid	0.241	**0.803**	**0.867**	**0.308**	**0.819**	−0.093	**0.507**	**0.577**	**0.509**	**0.534**	0.244	0.319	−0.026	−0.100	**0.577**	**0.845**				
Ratio piceid	**0.281**	0.114	**0.676**	**0.908**	0.170	−0.031	−0.019	**0.330**	**0.830**	0.056	0.278	0.551	−0.136	0.145	−0.145	0.029	**0.498**			
Sum piceid	0.079	**0.904**	**0.677**	−0.095	**0.898**	−0.119	**0.602**	**0.491**	0.180	**0.597**	0.095	0.094	0.059	−0.151	**0.785**	**0.996**	**0.875**	0.089		
Shikimic acid	0.053	−0.047	−0.076	−0.003	−0.051	**0.325**	−0.014	−0.108	−0.101	−0.008	−**0.338**	0.065	−0.147	−0.032	−0.154	−0.157	−0.212	−0.087	−0.162	
*trans*-Resveratrol	−0.066	**0.751**	**0.400**	−**0.372**	**0.722**	−0.177	**0.599**	**0.435**	0.006	**0.585**	−0.055	−**0.257**	−0.028	−**0.284**	**0.963**	**0.850**	**0.625**	−0.158	**0.830**	−0.162

Given are the correlation coefficients, significant correlations are shown in bold. For details see “Materials and methods” section

The concentrations of several compounds were highly correlated to each other (Table 3), most of them positively. The highest correlation coefficients were found within stilbenes, where the compounds astringin, piceid, isorhapontin, resveratrol and piceatannol were positively correlated to each other, and within the *cis*- and *trans*-forms and arithmetic parameters of each compounds. Similarly, the flavonoids were correlated to each other; kaempferol 3-glucoside and catechin in addition with shikimic acid.

### Population structure

**Fig. 1:**
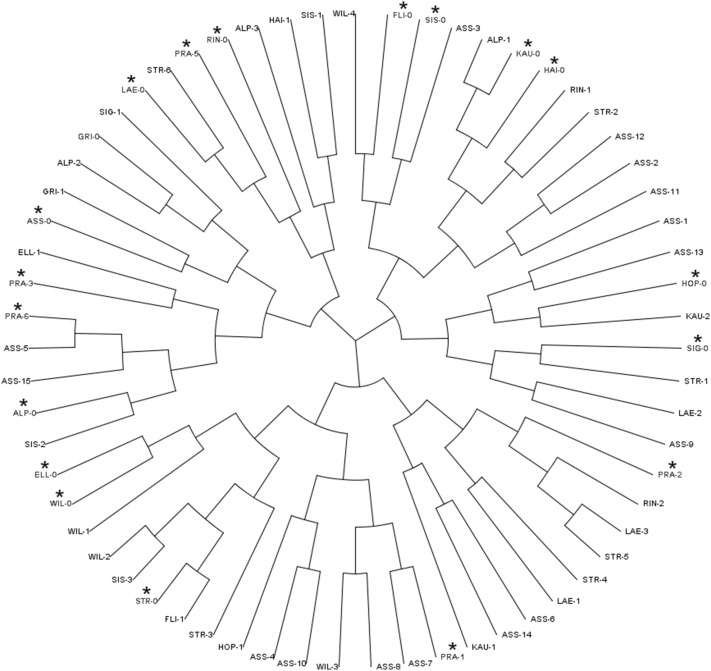
Cladogram built with 356 SNP markers using neighbour joining as clustering method. Trees with high susceptibility (with *asterisk*) and trees with low susceptibility show no grouping

When considering tree sampling locations as prior in population structure analysis, the likelihood (LnP) of K decreased with increasing K without reaching a continuous plateau that would be expected in the presence of a genetic structure (Supplemental Figure S1a). The number of genetic groups was also investigated with Evanno’s method, where the Delta K plot showed low values in all K tested (Fig. S1a). Bar plots demonstrated that all individuals are admixed and none of them was clearly assigned to one group. The alternative scenario, without considering sampling locations, revealed similar results (Fig. S1b), suggesting that the most probable K is one. This conclusion is reinforced by the PCA using SNP data (Supplemental Figure S2), where no group was clearly defined. Finally, the cladogram showed that individuals with high and low susceptibility are evenly distributed (Fig. 1).

### Association analysis model choice

**Fig. 2:**
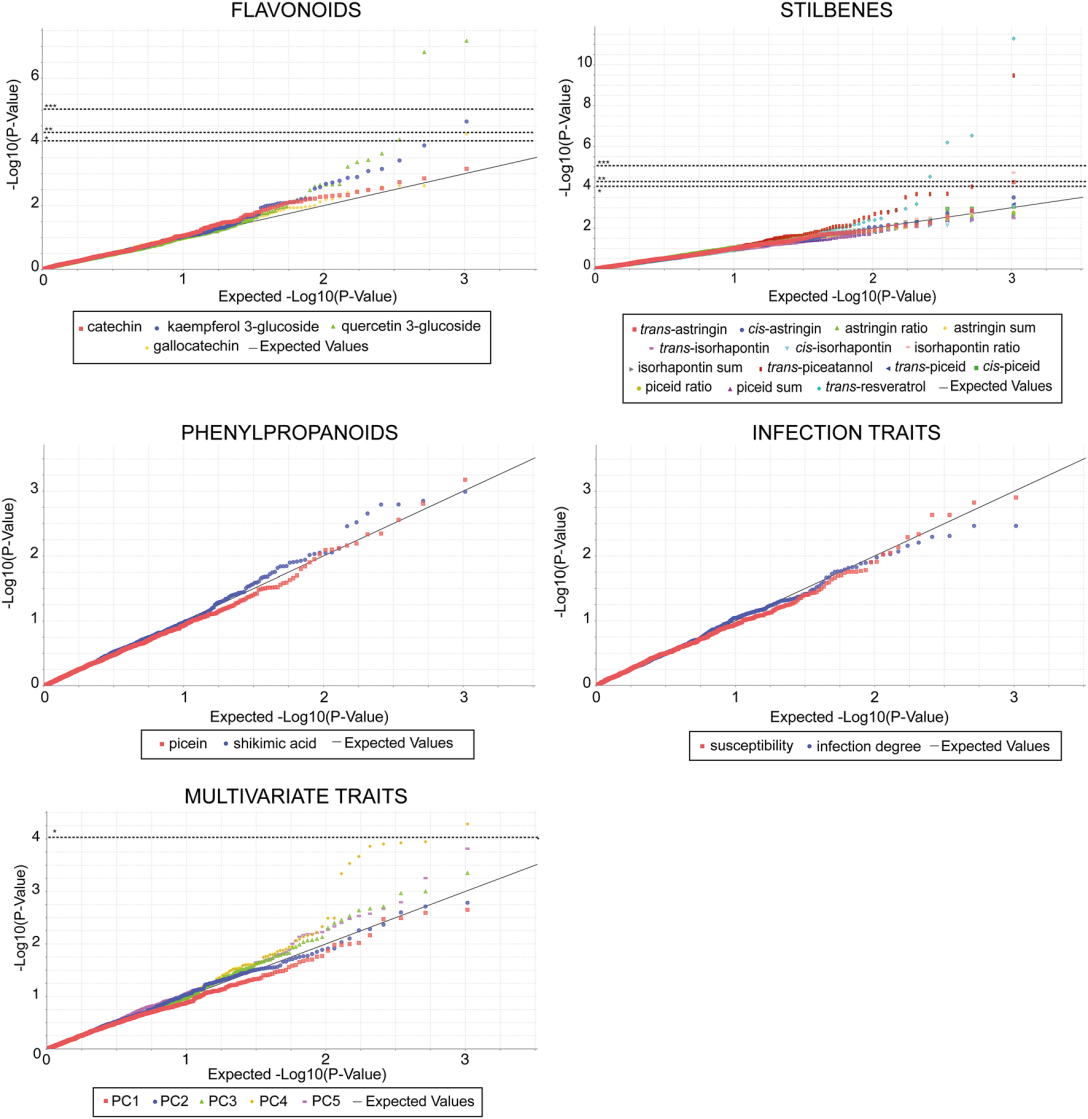
Q-Q plots for each of the GLM tests in all chemical, susceptibility and multivariate traits. *Horizontal dotted lines* indicate the Bonferroni corrected threshold for multiple test, ***p < 0.01; **p < 0.05; *p < 0.1

As STRUCTURE (Q) and PCA (P) did not show any structure in our population, models correcting for population stratification estimated by both methods are not presented and the GLM model without correction was selected for association analysis. Q-Q plots (Fig. 2) show the quality of the model for each single and multivariate trait. The GLM performed well for all traits, and no substantial deviations from the identity line were found, underlining our assumption of an unstructured population. Some markers with the lowest p values are lower than expected as a true association can be underneath.

### Association results

With the GLM model, 1035 SNPs (the complete genotype dataset is given in Supplementary Table S6) were tested against 20 chemical traits (stilbenes: *cis*-astringin, *trans*-astringin, astringin ratio, astringin sum, *cis*-isorhapontin, *trans*-isorhapontin, isorhapontin ratio, isorhapontin sum, *cis*-piceid, *trans*-piceid, piceid ratio, piceid sum, *trans*-piceatannol, *trans*-resveratrol; flavonoids: kaempferol 3-glucoside, quercetin 3-glucoside, catechin, gallocatechin; simple phenylpropanoids: shikimic acid, picein), two infection traits (infection degree, susceptibility) and five multivariate traits (PC1, PC2, PC3, PC4, PC5). For the latter, the first five principal components with the highest eigenvalues (Supplemental Table S4) were used (compare Beaulieu et al. 2011). Corresponding factor loadings are shown in Supplemental Table S5.

Among the 27,945 marker-trait pairs, 15 significant associations were found under the strictest conditions with a Bonferroni corrected threshold of 9.66 × 10^−5^ (p < 0.1). Furthermore, 31 significant associations were found under a q-value threshold of 10%. A total number of 20 markers were associated with the studied traits, and some of them were shared by different traits (Table 4). Most of the associations were found within stilbenes and flavonoids, 13 and 10 respectively, and from all the studied traits eight had markers significantly associated (gallocatechin, kaempferol 3-glucoside, quercetin 3-glucoside, *trans*-astringin, astringin sum, isorhapontin ratio, *trans*-resveratrol, *trans*-piceatannol, PC4). No associations were found for simple phenylpropanoids and 8 weak associations were found in just one multivariate trait (PC4); four of these weak associations were also found in flavonoid traits such as kaempferol 3-glucoside and quercetin 3-glucoside.

**Table 4:** Significant genetic associations

Trait	Marker name^a^		p value^b^		Rsq_marker^c^	q value^d^	
Flavonoids							
Gallocatechin	GQ03011-J06.1.333		5.56E–05	*	0.2786	5.75E–02	*
Kaempferol 3-glucoside	MA_28980g0010-2680-[T_G]	1, D	2.29E–05	**	0.3038	2.01E–02	***
Kaempferol 3-glucoside	GQ03121-E24.1.494	2	1.29E–04	NS	0.2580	5.68E–02	*
Quercetin 3-glucoside	CDF1-20-1232	A	6.69E–08	***	0.4288	6.21E–05	******
Quercetin 3-glucoside	MA_10267291g0010-503-[T_C]	B	1.52E–07	***	0.4292	7.04E–05	******
Quercetin 3-glucoside	PGLM2-1119		8.45E–05	*	0.2685	2.61E–02	**
Quercetin 3-glucoside	GQ0015-B03.1.189	3	2.31E–04	NS	0.2508	5.36E–02	*
Quercetin 3-glucoside	MA_121267g0010-916-[T_C]	C	3.70E–04	NS	0.2316	6.70E–02	*
Quercetin 3-glucoside	MA_28980g0010-2680-[T_G]	1, D	4.33E–04	NS	0.2309	6.70E–02	*
Quercetin 3-glucoside	GQ03121-E24.1.494	2	6.01E–04	NS	0.2190	7.97E–02	*
Stilbenes							
Isorhapontin ratio	PBB-PF00847-11-1		1.91E–05	**	0.3170	1.97E–02	***
*trans*-Astringin	GQ0015-B03.1.189	3	5.55E–05	*	0.2867	5.35E–02	*
Astringin sum	GQ0015-B03.1.189	3	5.60E–05	*	0.2865	5.35E–02	*
*trans*-Resveratrol	NODE-60-length-1132-cov-157.795929-418	4	5.22E–12	***	0.5917	5.21E–09	******
*trans*-Resveratrol	MA_57678g0010-2862-[G_T]	5	2.93E–07	***	0.4047	1.46E–04	*****
*trans*-Resveratrol	FCL3066Contig1-663	6	6.38E–07	***	0.3885	2.13E–04	*****
*trans*-Resveratrol	PabiesPRR1-240		3.02E–05	**	0.2932	7.53E–03	****
*trans*-Piceatannol	NODE-60-length-1132-cov-157.795929-418	4	3.35E–10	***	0.5287	2.93E–07	******
*trans*-Piceatannol	GQ03410-O07.1.1386		9.29E–05	*	0.2662	3.79E–02	**
*trans*-Piceatannol	NODE-966-length-765-cov-27.526798-686		2.07E–04	NS	0.2536	3.79E–02	**
*trans*-Piceatannol	MA_57678g0010-2862-[G_T]	5	2.15E–04	NS	0.2526	3.79E–02	**
*trans*-Piceatannol	FCL3066Contig1-663	6	2.16E–04	NS	0.2525	3.79E–02	**
*trans*-Piceatannol	GQ04111-M21.1.912		3.08E–04	NS	0.2470	4.49E–02	**
Multivariate traits							
PC4	GQ0176-C06.1.313		5.20E–05	*	0.2802	2.67E–02	**
PC4	CDF1-20-1232	A	1.13E–04	NS	0.2651	2.67E–02	**
PC4	MA_10267291g0010-503-[T_C]	B	1.19E–04	NS	0.2759	2.67E–02	**
PC4	MA_121267g0010-916-[T_C]	C	1.25E–04	NN	0.2588	2.67E–02	**
PC4	GQ03806-O09.1.555		1.38E–04	NS	0.2564	2.67E–02	**
PC4	NODE-10691-length-922-cov-115.009758-679		2.16E–04	NS	0.2563	3.48E–02	**
PC4	MA_28980g0010-2680-[T_G]	D	2.93E–04	NS	0.2410	4.04E–02	**
PC4	GQ03321-E07.1.796		4.58E–04	NS	0.2261	5.53E–02	*

^a^Superscript numbers (1–6) show markers associated to several traits, superscript letters (A–D) show markers associated to a multivariate trait

^b^Bonferroni corrected threshold for multiple test, ***p value <0.01; **p value <0.05; *p value <0.1; *NS* not statistically significant

^c^Rsq_marker, total explained phenotypic variation

^d^******q-value <1e–04; *****q-value <0.001; ****q-value <0.01; ***q-value <0.025; **q-value <0.05; *q-value <0.1

Concerning stilbenes, a total of six markers were associated with *trans-*piceatannol (R^2^ = 0.52–0.24), three of which (NODE-60-length-1132-cov-157.795929-418; MA_57678g0010-2862-[G_T]; FCL3066Contig1-663) were also associated with *trans*-resveratrol that exhibited a total of four associated markers (R^2^ = 0.59–0.29). One marker was linked to isorhapontin ratio (PBB-PF00847-11-1; R^2^ = 0.3170), and another one was associated with *trans*-astringin and shared by astringin sum (GQ0015-B03.1.189; R^2^ = 0.28), the latter being associated with the flavonoid quercetin 3-glucoside (R^2^ = 0.25). For flavonoids, seven markers were associated with quercetin 3-glucoside (R^2^ = 0.42–0.21), two of which (MA_28980g0010-2680-[T_G]; GQ03121-E24.1.494) were also associated with kaempferol 3-glucoside (R^2^ = 0.30–0.25). In addition, one SNP marker was associated with gallocatechin (GQ03011-J06.1.333; R^2^ = 0.27).

**Fig. 3:**
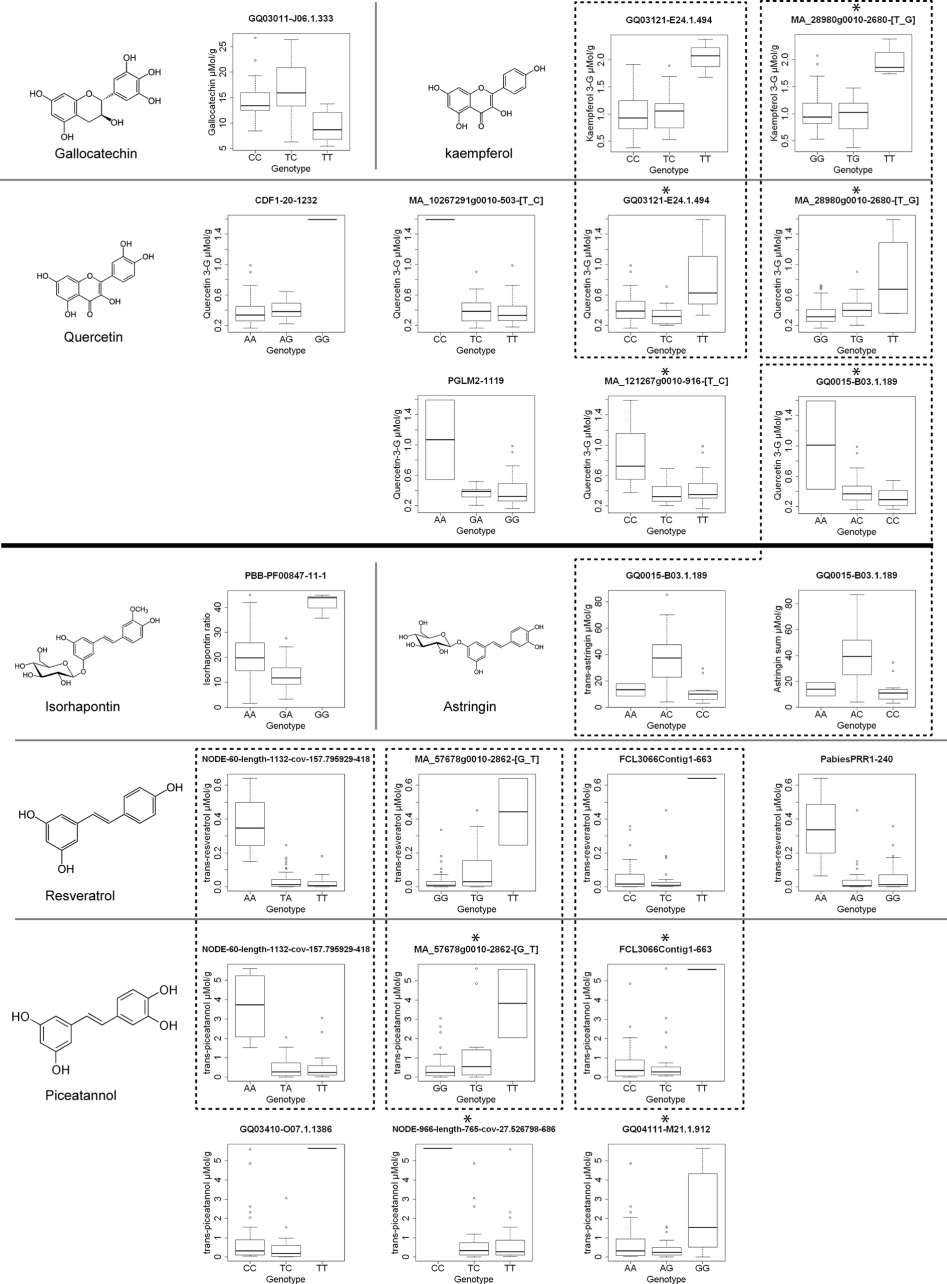
Box plot of flavonoids and stilbene concentrations or ratios by genotypes of associated SNP markers. The *boxes* represent the median (*black middle line*) limited by the 25th (Q1) and 75th (Q3) percentiles. *Dots* represent outliers. *Dotted lines* frame traits associated to the same marker. *Asterisks* indicate markers significantly associated below the q-value

**Fig. 4:**
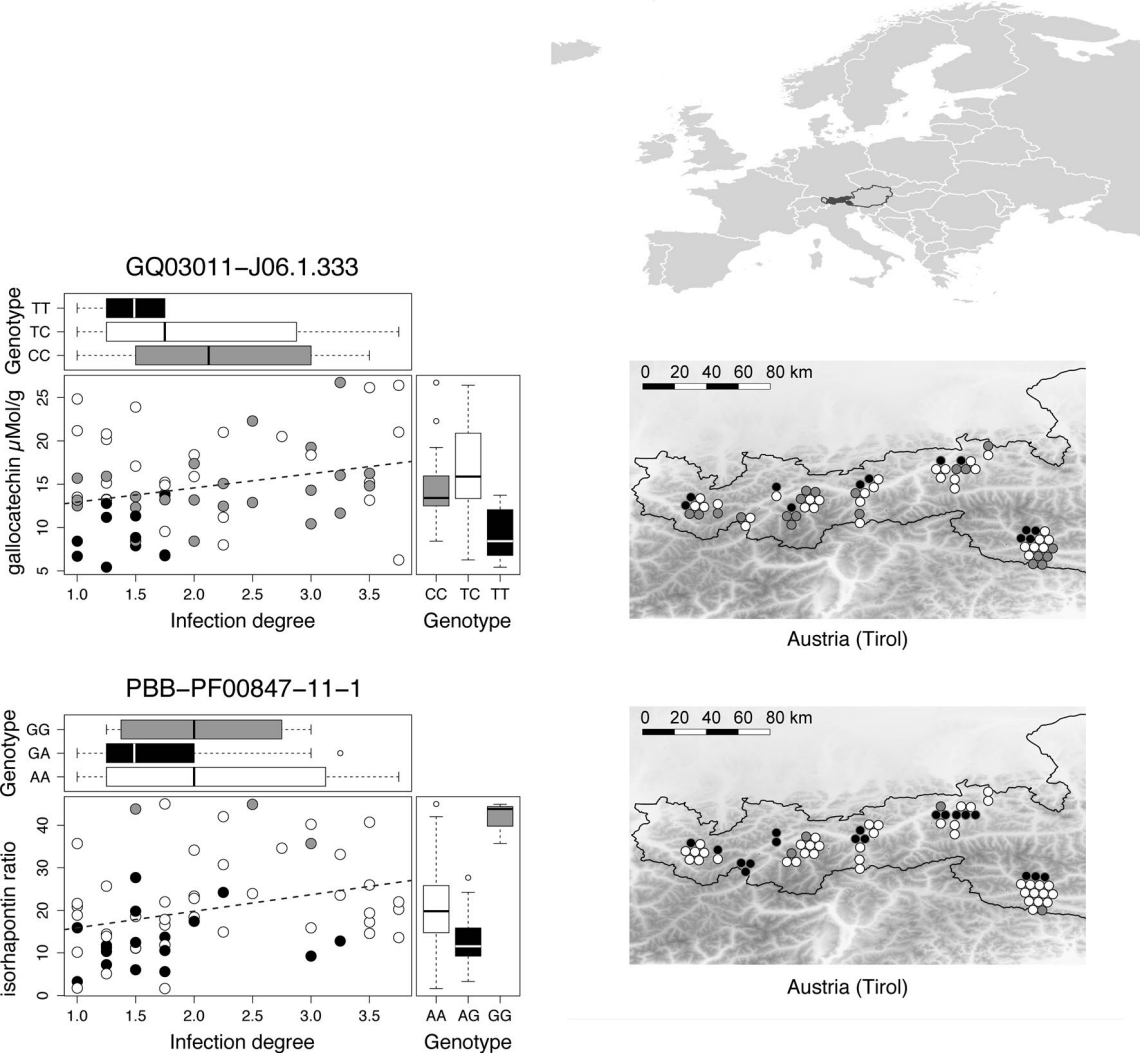
Scatterplot of gallocatechin and isorhapontin ratio vs. infection degree with marginal boxplots of the associated marker genotypes of GQ03011-J06.1.333 and PBB-PF00847-11-1 (*left*) and genotypes plotted in a location map (map projection, WGS1984) where all individuals are represented (*right*). *Dashed lines* represent linear regressions. Genotypes were coloured based on the highest (*grey*), lowest (*black*) and most variable (*white*) values for gallocatechin concentration and isorhapontin ratio. *Boxes* within the marginal boxplots represent the median (*black middle line*) limited by the 25th (Q1) and 75th (Q3) percentiles and *dots* represent outliers

Within each associated marker, chemical concentrations or ratios differed between allelic variants (Fig. 3). No significant genetic marker for the degree of infection or high and low susceptibility was detected, but the scatterplots of gallocatechin and isorhapontin ratio vs infection degree with marginal boxplots of the associated marker genotypes (Fig. 4a) revealed that some specific genotypes seem to have both the lowest infection degree and chemical concentration (gallocatechin − TT ^GQ03011-J06.1.333^) or ratio (isorhapontin ratio − GA ^PBB-PF00847-11-1^). Both markers are widely distributed in the studied population (Fig. 4b).

Markers significantly associated were mainly located within exons (14 markers). Others (5 markers) belong to introns, downstream regions or gene-empty regions (Table 5). Variation in seven exon-located markers affects the amino acid sequence (nonsynonymous substitution; missense change) as they are located mainly in the 1st or 2nd position of a codon. Nevertheless, variation in the remaining seven exon-located markers does not affect the amino acid sequence (synonymous substitution) as they are located mainly in the 3rd position of a codon.

**Table 5:** Characteristics of markers significantly associated

Marker name	Associated trait	Genomic position	Nucleotide	Exon /intron	Strand	SNP	SNP type	Codon position	Non synonymous substitution missense change	aa characteristics	PFAM description	GO terms
GQ03011-J06.1.333	gallocatechin	MA_10434464g0010	1786	Exon 1	+	[T/C]	NSS-M	2	ATT = I (isoleucine)/ACT = T (threonine)	I big and aliphatic-hydrophobic; T small and polar	Mitochondrial carrier protein	P: mitochondrial transport
												F: transporter activity
												C: plastid
												C: mitochondrial inner membrane
MA_28980g0010-2680-[T_G]	Kaempferol 3-glucoside; quercetin 3-glucoside; PC4	MA_28980g0010	31,964	Intron	+	[T/G]	UTR				Zinc finger, C_3_HC_4_ type (RING finger)	**
											Copine	
											RING-type zinc-finger, LisH dimerisation motif	
GQ03121-E24.1.494	Kaempferol 3-glucoside; quercetin 3-glucoside	MA_6741659g0010	1238	Exon 2	+	[T/C]	SS	3	CG[T/C] = R (arginine)		*	**
CDF1-20-1232	Quercetin 3-glucoside; PC4	MA_2430g0010	9488	Exon 1	−	[A/G]	NSS-M	1	ACC = T (threonine)/GCC = A (alanine)	Both small and polar	*	**
MA_10267291g0010-503-[T_C]	Quercetin 3-glucoside; PC4	MA_10267291g0010	759	Exon 3	+	[T/C]	SS	1	[T/C]TG = L (leucine)		Fascin domain	**
											Ricin-type beta-trefoil lectin domain-like	
PGLM2-1119	Quercetin 3-glucoside	MA_79559g0010	1385	Exon 2	+	[A/G]	SS	3	CT[A/G] = L (leucine)		Histidine kinase-, DNA gyrase B-, and HSP90-like ATPase	P: cell differentiation
												F: nucleotide binding
												F: kinase activity
GQ0015-B03.1.189	Quercetin 3-glucoside; *trans*-astringin; astringin sum	MA_804816g0010	1531	Exon 1	−	[A/C]	NSS-M	2	TCT = S (serine)/TAT = Y (tyrosine)	S small and polar; Y big and aromatic	Leucine rich repeat	**
											NB-ARC domain	
											Leucine Rich repeats (2 copies)	
MA_121267g0010-916-[T_C]	Quercetin 3-glucoside; PC4	MA_121267g0010	4241	Exon 1	+	[T/C]	NSS-M	1	TGT = C (cysteine)/CGT = R (arginine)	C small; R charged positive	Tetratricopeptide repeat	**
											PPR repeat	
											PAAD/DAPIN/Pyrin domain	
											Bacterial transcriptional activator domain	
											Protein of unknown function (DUF1686)	
											26 S proteasome subunit RPN7	
											PPR repeat family	
											Pentatricopeptide repeat domain	
PBB-PF00847-11-1	Isorhapontin ratio	MA_37766g0010	1544	Exon 2	+	[A/G]	SS	3	TC[A/G] = S (serine)		AP2 domain	**
NODE-60-length-1132-cov-157.795929-418	*trans*-Resveratrol; *trans*-piceatannol	No gene—no closer gene in 1 kb (MA_184576g0010)	18,345		+	[T/A]	UTR					
MA_57678g0010-2862-[G_T]	*trans*-Resveratrol; *trans*-piceatannol	MA_57678g0010	6415	Intron	+	[T/G]	UTR				Cytochrome P450	**
FCL3066Contig1-663	*trans*-Resveratrol; *trans*-piceatannol	No gene—downstream (47 nt) MA_86404g0010	1461		+	[T/C]	UTR				Ribosomal L5P family C-terminus	P: cysteine biosynthetic process
												P: isopentenyl diphosphate biosynthetic process, mevalonate-independent pathway
												P: translation
												F: structural constituent of ribosome
												C: membrane
PabiesPRR1-240	*trans*-Resveratrol	MA_71728g0010	12,142	Exon 7	−	[A/G]	SS	3	TC[A/G] = S (serine)		Response regulator receiver domain	P: cellular process
											CCT motif	
GQ03410-O07.1.1386	*trans*-Piceatannol	No gene—downstream (56 nt) MA_865616g0010	3820		+	[T/C]	UTR				Vacuolar protein sorting-associated protein 26	P: vacuolar transport
												C: retromer complex
NODE-966-length-765-cov-27.526798-686	*trans*-Piceatannol	MA_105331g0010	1788	Exon 1	+	[T/C]	NSS-M	2	ATG = M (methionine)/ACG = T (threonine)	M big and hydrophobic; T small and polar	*	**
GQ04111-M21.1.912	*trans*-Piceatannol	MA_10428089g0010	12,081	Exon 5	+	[A/G]	NSS-M	2	AAA = K (lysine)/AGA = R (arginine)	Both charged positive	*	**
GQ03806-O09.1.555	PC4	MA_8052g0010	42,129	Exon 4	+	[T/C]	SS	3	AA[T/C] = N (asparagine)		*	**
NODE-10691-length-922-cov-115.009758-679	PC4	Unknown	Unknown	Unknown	Unknown	[A/G]	unknown					
GQ03321-E07.1.796	PC4	MA_533908g0010	7681	Exon 4	+	[A/C]	SS	3	GC[A/C] = A (alanine)		GDSL-like lipase/acylhydrolase	**
GQ0176-C06.1.313	PC4	MA_10437174g0010	45,470	Exon	−	[T/A]	NSS-M	2	AAT = N (asparagine)/ATT = I (isoleucine)	N polar; I nonpolar and aliphatic	*	**

*SS* synonymous substitution, *NSS*-*M* non synonymous substitution Missense change, *UTR* untranslated regions, *P* biological process, *F* molecular function, *C* cellular component

*PFAM ID could not be found

**Gene Ontology values could not be found

Within all contigs, linkage disequilibrium decreased rapidly with physical distance (Supplemental Figure S3). However, within individual genes and contigs, LD showed strong heterogeneity as shown in the Fig. S3b, c in agreement with previous studies (Pavy et al. 2012).

## Discussion

This is one of the so far very few available studies identifying DNA sequence variants correlated with concentration levels of secondary metabolites and pathogen susceptibility in a coniferous forest tree species. The important preconditions for association mapping, a population that lacks genetic structure and large phenotypic variability, were fulfilled. A total of 31 trait associations were found, explaining 22–59% of phenotypic variation, and several markers were shared by different traits. The phenolics profile of healthy needles differed between trees showing high and low susceptibility to the fungus.

### Metabolic characteristics

#### Different phenolic composition of trees showing high and low susceptibility

Trees with low susceptibility to *C. rhododendri* showed statistically significant lower concentrations of the compounds gallocatechin and *cis*-piceid as well as lower *cis*/*trans*-ratios of astringin, isorhapontin and piceid in the needles compared to highly susceptible trees (see Table 2). Higher levels of piceid were found also in Norway spruce susceptible to *E. polonica* (Brignolas et al. 1995) and in cultivars of grapevine susceptible to downy mildew compared to resistant plants (Pezet et al. 2004). The higher levels of phenolic compounds in trees challenged with the fungus could be based on an induced systemic accumulation of phenolics due to repeated infection, as found for example in spruce infected by *Sirococcus conigenus* (Bahnweg et al. 2000), *E. polonica* (Brignolas et al. 1995; Evensen et al. 2000; Krajnc et al. 2014) or *Heterobasidion* spp. (Danielsson et al. 2011). Contrariwise, trees showing low susceptibility may incorporate soluble phenolics into the cell wall to isolate the biotrophic fungus (Matern and Kneusel 1988; Fossdal et al. 2012) by preventing nutrient uptake by haustoria in the initial infection phase, thereby avoiding the development of disease symptoms. In addition, they may activate a rapid modification of phenolics by isomerization, de-glycosylation, methoxylation or oligomerization (Chong et al. 2009), resulting in higher concentrations of the active form. Direct toxicity of hydroxystilbenes can be related to the capacity of metabolites to disrupt cell membranes, nuclear and mitochondrial membranes in fungal germ tubes (Pezet and Pont 1990). Furthermore, compounds may confer resistance as a group by synergy effects (Wallis et al. 2008) and the variability of the phenolic composition in time and space may challenge the fungus, like similarly suggested for leaf-eating invertebrates (Edenius et al. 2012).

#### Extraordinary high concentrations of several compounds in individual trees

Several analysed trees showed extraordinary high levels of individual compounds, among them piceatannol (5.63 µMol g^−1^), resveratrol (0.64 µMol g^−1^) and quercetin 3-glucoside (1.59 µMol g^−1^, compare also maxima values in Table 2). As phenolic secondary metabolites mediate interactions with several pathogenic and herbivory organisms, these extreme phenotypes could be candidate trees for future experiments and forestation strategies. Furthermore, due to the broad pharmacological effects and difficult chemical synthesis of these compounds (see e.g. Lin and Yan 2014) the use of spruce material for extraction and economic exploitation could be considered. For example, *trans*-resveratrol was produced by the tree GRI-O in extraordinarily high amounts (0.64 µMol g^−1^ dried needle tissue), compared to grapes (0.22–0.44 µMol g^−1^) and red wine (up to 0.07 µMol ml^−1^), which are considered major sources of resveratrol (Lekli et al. 2010).

#### Correlations between compound concentrations

High correlations between the concentrations of individual phenolic compounds (see Table 3) reflect similarities of the chemical structures and shared steps in the metabolic pathway (compare Hammerbacher et al. 2011; Laboratories 2013). These findings are also reflected by shared genetic markers of related compounds (see Table 4).

### Population structure

In Europe, Norway spruce genetic resources exhibit differences mainly between the Baltico-Nordic and the Alpine domain, while genetic differentiation among populations within these domains was found to be low (Heuertz et al. 2006). In particular, this is true for the central and western alpine range of Norway spruce, which was found to originate from a single phylogeographic lineage mainly (Gugerli et al. 2001; Tollefsrud et al. 2008). Our present analysis with individuals from the central Tyrolean Alps confirms the low genetic structure as no stratification could be found for 356 putatively neutral SNPs. This high number of SNPs was used to avoid ascertainment bias that could induce cryptic population structure (Lachance and Tishkoff 2013). Also, accounting for the geographic location of the trees (see Fig. S1a) did not affect the association results, as shown for other studies with weak population structure signals (Porras-Hurtado et al. 2013). Therefore, the association study could be done without taking any structure into account and the risk for detection of false positives was low. Furthermore, cladogram and PCA underlined that groups of trees showing high and low susceptibility did not belong to genetically related groups and do not originate from one common ancestor or a certain provenance (see Fig. 1), but that lower susceptibility occurs broadly across various locations and populations. A similar model was already explored by Jourdan et al. (2015) in an unstructured population. McKown et al. (2014) found in a *P. trichocarpa* association population that in all cases the simple P or Q models were sufficient and no traits required the more stringent K, Q + K or P + K models. Thus, McKown et al. avoided overcorrection by more stringent models that control for stratification such as MLM (Allwright et al. 2016). This is very comparable to our case, were no structure could be detected by several methods.

### Significant genetic markers

Thirty-one significant genetic associations for phenolic compounds were identified, yet none for the traits susceptibility or infection degree that were both based on the phenotypic assessment in the field (see Table 4). The latter may be partly due to the difficult assessment of field susceptibility, which might be affected by local spore densities and weather conditions (Ganthaler and Mayr 2015), thus obscuring more direct relations of phenotypes showing low susceptibility to the set of SNP markers tested. However, respective gallocatechin, genotypes TT ^GQ03011−J06.1.333^ seemed to have both the lowest infection degree and chemical concentrations (see Fig. 4).

Complex traits, including phytochemicals and quantitative disease resistance, are usually regulated by many genes with small and additive effects and associated markers span coding as well as non-coding portions of genes (Gonzalez-Martinez et al. 2007; Quesada et al. 2010; Eckert et al. 2012). Accordingly, several genetic markers for stilbene and flavonoid concentrations were found and some were associated with several compounds (compare Table 4), likely due to the complexity and interconnection of phenolic pathways (Vogt 2010). In order to capture these correlations and complexity, multivariate traits were constructed by PCA, such as successfully done for other complex traits in conifers (González-Martínez et al. 2007; Eckert et al. 2009). As expected, most of the markers associated with PC4 were also associated with its main loading factors kaempferol 3-glucoside and quercetin 3-glucoside. However, shikimic acid had a negative contribution, as this product is upstream of the main flavonoid synthetic pathway (Vogt 2010). Genetic control by many loci is connected with relatively small individual effects (phenotypic variation R^2^ explained by markers), similar or partly higher compared to other conifer studies (Table 4; González-Martínez et al. 2008; Eckert et al. 2009, 2012; Beaulieu et al. 2011). There are indications that in conifers stilbene synthesis is based on multiple copies of the same genes that are under the control of different promoters and can be regulated in response to different internal and external factors (Hammerbacher et al. 2011). Therefore, analysis is complicated by the influence of both genetic and environmental factors on metabolites (Fiehn 2002).

Significant SNPs were located in coding as well as non-coding regions, with partly synonymous and nonsynonymous polymorphisms (see Table 5). Markers located in untranslated regions (introns, downstream regions or gene-empty regions) should also be considered due to their possible impact on gene expression through differential transcription, siRNA targeting or mRNA stability (Webb et al. 2009). Therefore, most of the SNPs are not directly responsible for the phenotypic variation but probably in LD with the causative change. To better understand the molecular function of marker linked genes, we examined their P-FAM domains. Pleiotropy could explain the diversity of genes detected, as most genes of interest are putatively involved upstream of the defence pathways and could enhance, modify or disrupt chemical profiles (Porth et al. 2014).

#### Signal transduction

Marker PGLM2-1119 associated with quercetin 3-glucoside is located on the gene MA_79559g0010 with a GHKL domain (Gyrase, Hsp90, Histidine Kinase, MutL), an evolutionary conserved protein domain (Dutta and Inouye 2000) that represents the structurally related ATPase domains of histidine kinase, DNA gyrase B and HSP90. Protein kinases play a central role in signalling during pathogen recognition and the subsequent activation of plant defence mechanisms (Romeis 2001). Moreover, marker PabiesPRR1-240 associated with *trans*-resveratrol is located in MA_71728g0010, a gene that could be involved in the defence pathway as a response regulator.

#### Transcriptional regulation

Transcription factors (TFs) are key players in plant innate immunity and activation of defence pathways. Marker GQ0015-B03.1.189, associated with both flavonoids and stilbenes, is located in the gene MA_804816g0010 that encodes a protein with a leucine-rich repeat (LRR) domain, frequently involved in plant defence (Shanmugam 2005; Tameling et al. 2006). In our study a non-synonymous substitution results in an amino acid change from S (small and polar) to Y (big and aromatic), potentially altering the phenotype. Seven related *Arabidopsis thaliana* disease resistance protein genes (AT1G33560; AT5G66900; AT4G33300; AT5G04720; AT5G47280; AT5G66910; AT5G66890; AT3G26470) make this candidate interesting. Marker PBB-PF00847-11-1, associated with isorhapontin ratio, is located in the coding sequence of MA_37766g0010, a putative member of the Apetala2/Ethylene-responsive element-binding protein family (AP2/EREBP). In *A. thaliana*, ethylene response factors (ERF) are directly responsible for the transcriptional regulation of several jasmonate/ethylene-responsive defence genes (Pré et al. 2008). In our study, GA ^PBB-PF00847-11-1^ genotypes seem to have both the lowest percentage of infected needles and isorhapontin *cis*/*trans*-ratio (see Fig. 4). Although these allelic variants represent a synonymous mutation, transcription, splicing, mRNA transport, and translation could be affected, possibly altering the phenotype and rendering the synonymous mutation non-silent (Goymer 2007).

#### Modification of compounds

One of the most promising markers found is MA_57678g0010-2862-[G_T], located in an intron of a gene belonging to the cytochrome P450 superfamily, the largest enzymatic protein family in plants and key players in plant development and defence (Xu et al. 2015). They are involved in multiple metabolic pathways and are important for breeding and biotechnology due to their capacity to modify and activate diverse secondary metabolites with ecological and pharmacological properties, including most terpenes, flavonoids and alkaloids (Villa-Ruano et al. 2015). In our study, *trans*-resveratrol and *trans*-piceatannol production were associated with this gene.

## Conclusions

Association mapping of forest trees in their natural environment enables a deeper understanding of genetic adaptation, despite the complex genetic architecture of most analysed traits. This has potentially important implications for plant material selection, for example for high-alpine afforestations and climate change adaptation strategies. Considering the broad ecological function of these compounds (compare Levin 1971), further research will benefit from the genetic knowledge gained. Results are likely to be useful in molecular marker-assisted selection and breeding for enhanced resistance and phytochemical production by increasing selection intensity, identifying more trees with low susceptibility, reducing the breeding cycle and overcoming temporal impediments such as age to trait expression. However, markers should be validated at least for the Alpine populations to exclude non-stable MTAs and reduce the risk of a break down with successive rounds of sexual reproduction and recombination. The present study utilized SNP markers that were found to be variable within Norway spruce and related conifers. Rare point mutation that could potentially cause pathogen resistance might have been withdrawn in this selection procedure. To date, the applied SNP array was the most affordable tool to explore the genetic background of the studied phenotypes. Further exploration should make use of a candidate gene approach or a high coverage of the whole genome. Moreover, further studies may benefit from a direct determination of the trait susceptibility and genotype environment interactions, for example by controlled inoculation tests on genetically identical clonal cuttings (Neale and Savolainen 2004), and the consideration of variations in the concentration of phenolic compounds during needle development.

## Electronic supplementary material

Below is the link to the electronic supplementary material.

Supplementary material 1 (DOCX 23 KB)

Supplementary material 2 (XLSX 405 KB)

Supplementary material 3 (DOCX 1194 KB)

Supplementary material 4 (TXT 183 KB)

Supplementary material 5 (TXT 17 KB)

## References

[CR1] Allwright M, Payne A, Emiliani G (2016). Biomass traits and candidate genes for bioenergy revealed through association genetics in coppiced European *Populus nigra* (L.). Biotechnol Biofuels.

[CR2] Andrew RL, Wallis IR, Harwood CE, Henson M, Foley WJ (2007). Heritable variation in the foliar secondary metabolite sideroxylonal in *Eucalyptus* confers cross-resistance to herbivores. Oecologia.

[CR3] Bahnweg G, Schubert R, Kehr RD, Müller-Starck G, Heller W, Langebartels C, Sandermann H (2000). Controlled inoculation of Norway spruce (*Picea abies*) with *Sirococcus conigenus*: PCR-based quantification of the pathogen in host tissue and infection-related increase of phenolic metabolites. Trees.

[CR4] Bailly C, Kranner I, Kermode AR (2011). Analyses of reactive oxygen species and antioxidants in relation to seed longevity and germination. Seed dormancy: methods and protocols. methods in molecular biology 773, springer protocols.

[CR5] Beaulieu J, Doerksen T, Boyle B (2011). Association genetics of wood physical traits in the conifer white spruce and relationships with gene expression. Genetics.

[CR6] Bernards MA, Båstrup-Spohr L, Schaller A (2008). Phenylpropanoid metabolism induced by wounding and insect herbivory. Induced plant resistance to herbivory.

[CR7] Bradbury PJ, Zhang Z, Kroon DE, Casstevens TM, Ramdoss Y, Buckler ES (2007). TASSEL: software for association mapping of complex traits in diverse samples. Bioinformatics.

[CR8] Brignolas F, Lacroix B, Lieutier F (1995). lnduced responses in phenolic metabolism in two Norway spruce clones after wounding and inoculations with *Ophiostoma polonicum*, a bark beetle-associated fungus. Plant Physiol.

[CR9] Brignolas F, Lieutier F, Sauvard D, Christiansen E, Berryman AA (1998). Phenolic predictors for Norway spruce resistance to the bark beetle *Ips typographus* (Coleoptera: Scolytidae) and an associated fungus, *Ceratocystis polonica*. Can J Forest Res.

[CR10] Budde KB, Heuertz M, Hernández-Serrano A, Pausas JG, Vendramin GG, Verdú M, González-Martínez SC (2014). In situ genetic association for serotiny, a fire-related trait, in Mediterranean maritime pine (*Pinus pinaster*). New Phytol.

[CR11] Burton P, Clayton D, Cardon L (2007). Genome-wide association study of 14,000 cases of seven common diseases and 3000 shared controls. Nature.

[CR12] Chan EKF, Rowe HC, Kliebenstein DJ (2010). Understanding the evolution of defense metabolites in *Arabidopsis thaliana* using genomewide association mapping. Genetics.

[CR13] Chen J, Källman T, Gyllenstrand N, Lascoux M (2010). New insights on the speciation history and nucleotide diversity of three boreal spruce species and a Tertiary relict. Heredity.

[CR14] Chen J, Källman T, Ma X (2012). Disentangling the roles of history and local selection in shaping clinal variation of allele frequencies and gene expression in Norway spruce (*Picea abies*). Genetics.

[CR15] Chen J, Uebbing S, Gyllenstrand N, Lagercrantz U, Lascoux M, Kallman T (2012). Sequencing of the needle transcriptome from Norway spruce (*Picea abies* Karst L.) reveals lower substitution rates, but similar selective constraints in gymnosperms and angiosperms. BMC Genom.

[CR16] Chen J, Källman T, Ma X, Zaina G, Morgante M, Lascoux M (2016). Identifying genetic signatures of natural selection using pooled population sequencing. Picea abies.

[CR17] Chong J, Poutaraud A, Hugueney P (2009). Metabolism and roles of stilbenes in plants. Plan Sci.

[CR18] Cumbie WP, Eckert A, Wegrzyn J, Whetten R, Neale D, Goldfarb B (2011). Association genetics of carbon isotope discrimination, height and foliar nitrogen in a natural population of *Pinus taeda* L. Heredity.

[CR19] Cvikrová M, Malá J, Hrubcová M, Eder J, Foretová S (2008). Induced changes in phenolic acids and stilbenes in embryogenic cell cultures of Norway spruce by culture filtrate of *Ascocalyx abietina*. J Plant Dis Prote.

[CR20] Danielsson M, Lundén K, Elfstrand M (2011). Chemical and transcriptional responses of Norway spruce genotypes with different susceptibility to *Heterobasidion* spp. infection. BMC Plant Biol.

[CR21] De Bary A (1879). Aecidium abietinum. Bot Z.

[CR22] Delvas N, Bauce É, Labbé C, Ollevier T, Bélanger R (2011). Phenolic compounds that confer resistance to spruce budworm. Entomol Exp Appl.

[CR23] Dillon SK, Nolan M, Li W, Bell C, Wu HX, Southerton SG (2010). Allelic variation in cell wall candidate genes affecting solid wood properties in natural populations and land races of *Pinus radiata*. Genetics.

[CR24] Dufrénoy J (1932). The unequal susceptibility of Spruces towards *Chrysomyxa rhododendri*. Comptes Rendus Soc Hebdom Soc Biol Filial.

[CR25] Dutta R, Inouye M (2000). GHKL, an emergent ATPase/kinase superfamily. Trends Biochem Sci.

[CR26] Earl DA, vonHoldt BM (2012). STRUCTURE HARVESTER: a website and program for visualizing STRUCTURE output and implementing the Evanno method. Conservation Genet Res.

[CR27] Eckert AJ, Bower AD, Wegrzyn JL (2009). Association genetics of coastal Douglas fir (*Pseudotsuga menziesii* var. *menziesii*, Pinaceae). I. Cold-hardiness related traits. Genetics.

[CR28] Eckert AJ, Wegrzyn JL, Cumbie WP (2012). Association genetics of the loblolly pine (*Pinus taeda*, Pinaceae) metabolome. New Phytol.

[CR29] Edenius L, Grzegorz M, Witzell J, Berghd J (2012). Effects of repeated fertilization of young Norway spruce on foliar phenolics and arthropods: implications for insectivorous birds’ food resources. Forest Ecol Manag.

[CR30] Evanno G, Regnaut S, Goudet J (2005). Detecting the number of clusters of individuals using the software STRUCTURE: a simulation study. Mol Ecol.

[CR31] Evensen PC, Solheim H, Hoiland K, Stenersen J (2000). Induced resistance of Norway spruce, variation of phenolic compounds and their effects on fungal pathogen. Forest Pathol.

[CR32] Falush D, Stephens M, Pritchard JK (2003). Inference of population structure using multilocus genotype data: linked loci and correlated allele frequencies. Genetics.

[CR33] Fiehn O (2002). Metabolomics: the link between genotypes and phenotypes. Plant Mol Biol.

[CR34] Fossdal CG, Nagy NE, Hietala AM, Kvaalen H, Slimestad R, Woodward S, Solheim H (2012). Indications of heightened constitutive or primed host response affecting the lignin pathway transcripts and phenolics in mature Norway spruce clones. Tree Physiol.

[CR35] Fuchs J, Kreiner M, Müller G, Oblasser H, Perle A, Riccabona F, Schwaninger C, Simon A, Stöhr D, Wallner M, Weber A, Zimmermann G (2016) Tiroler Waldbericht 2016. Amt der Tiroler Landesregierung, Gruppe Forst. http://www.tirol.gv.at/themen/umwelt/wald/zustand/waldzustandsbericht. Accessed 18 January 2017

[CR36] Ganthaler A, Mayr S (2015). Temporal variation in airborne spore concentration of *Chrysomyxa rhododendri*: correlation with weather conditions and consequences for Norway spruce infection. Forest Pathol.

[CR37] Ganthaler A, Bauer H, Gruber A, Mayr M, Oberhuber W, Mayr S (2014). Effects of the needle bladder rust (*Chrysomyxa rhododendri*) on Norway spruce: implications for subalpine forests. Eur J Forest Res.

[CR38] Gäumann E (1959) Die Rostpilze Mitteleuropas. Beitr. Kryptogamenflora der Schweiz 12, Verlag Büchler, Bern

[CR39] González-Martínez SC, Wheeler NC, Ersoz E, Dana Nelson C, Neale DB (2007). Association genetics in *Pinus taeda* L. I. Wood property traits. Genetics.

[CR40] González-Martínez SC, Huber D, Ersoz E, Davis JM, Neale DB (2008). Association genetics in *Pinus taeda* L. II. Carbon isotope discrimination. Heredity.

[CR41] Goymer P (2007). Synonymous mutations break their silence. Nat Rev Genet.

[CR42] Gugerli F, Sperisen C, Büchler U, Magni F, Geburek T, Jeandroz S, Senn J (2001). Haplotype variation in a mitochondrial tandem repeat of Norway spruce (*Picea abies*) populations suggests a serious founder effect during postglacial re-colonization of the western Alps. Mol Ecol.

[CR43] Guillet-Claude C, Isabel N, Pelgas B, Bousquet J (2004). The evolutionary implications of knox-I gene duplications in conifers: Correlated evidence from phylogeny, gene mapping, and analysis of functional divergence. Mol Biol Evol.

[CR44] Hakulinen J, Sorjonen S, Julkunen-Tiitto R (1999). Leaf phenolics of three willow clones differing in resistance to *Melampsora* rust infection. Physiol Plant.

[CR45] Hamilton JG, Zangerl AR, DeLucia EH, Berenbaum MR (2001). The carbon-nutrient balance hypothesis: its rise and fall. Ecol Lett.

[CR46] Hammerbacher A, Ralph SG, Bohlmann J, Fenning TM, Gershenzon J, Schmidt A (2011). Biosynthesis of the major tetrahydroxystilbenes in spruce, astringin and isorhapontin, proceeds via resveratrol and is enhanced by fungal infection. Plant Physiol.

[CR47] Hammerbacher A, Schmidt A, Wadke N (2013). A common fungal associate of the spruce bark beetle metabolizes the stilbene defenses of Norway spruce. Plant Physiol.

[CR48] Hammerschmidt R (2005). Phenols and plant-pathogen interactions: the saga continues. Physiol Mol Plant Pathol.

[CR49] Heer K, Ullrich KK, Liepelt S, Rensing SA, Zhou J, Ziegenhagen B, Opgenoorth L (2016). Detection of SNPs based on transcriptome sequencing in Norway spruce (*Picea abies* (L.) Karst). Conserv Genet Res.

[CR50] Heuertz M, De Paoli E, Källman T (2006). Multilocus patterns of nucleotide diversity, linkage disequilibrium and demographic history of Norway spruce [*Picea abies* (L.) Karst]. Genetics.

[CR51] Hjältén J, Niemi L, Wennström A, Ericson L, Roininen H, Julkunen-Tiitto R (2007). Variable responses of natural enemies to *Salix triandra* phenotypes with different secondary chemistry. Oikos.

[CR52] Holliday JA, Ritland K, Aitken SN (2010). Widespread, ecologically relevant genetic markers developed from association mapping of climate related traits in Sitka spruce (*Picea sitchensis*). New Phytol.

[CR53] Hubisz MJ, Falush D, Stephens M, Pritchard JK (2009). Inferring weak population structure with the assistance of sample group information. Mol Ecol Res.

[CR54] Jeandet P, Delaunois B, Conreux A (2010). Biosynthesis, metabolism, molecular engineering, and biological functions of stilbene phytoalexins in plants. Biofactors.

[CR55] Jourdan M, Gagne S, Dubois-Laurent C (2015). Carotenoid content and root color of cultivated carrot: a candidate-gene association study using an original broad unstructured population. PLoS One.

[CR56] Källman T, De Mita S, Larsson H (2014). Patterns of nucleotide diversity at photoperiod related genes in Norway spruce [*Picea abies* (L.) Karst.]. PLoS One.

[CR57] Kanehisa Laboratories (2013) Kyoto Encyclopedia of Genes and Genomes. http://www.genome.jp/kegg/pathway.html. Accessed 7 January 2016

[CR58] Keeling CI, Weisshaar S, Lin RPC, Bohlmann J (2008). Functional plasticity of paralogous diterpene synthases involved in conifer defense. P Natl Acad Sci USA.

[CR59] Krajnc AU, Novak M, Felicijan M, Kraševec N, Lešnik M, Zupanec N, Komel R (2014). Antioxidative response patterns of Norway spruce bark to low-density *Ceratocystis polonica* inoculation. Trees.

[CR60] Külheim C, Yeoh SH, Wallis IR, Laffan S, Moran GF, Foley WJ (2011). The molecular basis of quantitative variation in foliar secondary metabolites in *Eucalyptus globulus*. New Phytol.

[CR61] Lachance J, Tishkoff SA (2013). SNP ascertainment bias in population genetic analyses: why it is important, and how to correct it. Bioessays.

[CR62] Lekli I, Ray D, Das DK (2010). Longevity nutrients resveratrol, wines and grapes. Genes Nutr.

[CR63] Lepoittevin C, Harvengt L, Plomion C, Garnier-Géré P (2012). Association mapping for growth, straightness and wood chemistry traits in the *Pinus pinaster* Aquitaine breeding population. Tree Genet Genom.

[CR64] Levin DA (1971). Plant phenolics: an ecological perspective. Am Nat.

[CR65] Lieutier F, Brignolas F, Sauvard D et al (1997) Phenolic compounds as predictors of Norway spruce resistance to bark beetles. In: Grégoire JC, Liebhold AM, Stephen FM, Day KR, Salom SM (ed) Proceedings: Integrating cultural tactics into the management of bark beetle and reforestation pests. USDA Forest Service General Technical Report NE-236, pp 215–216

[CR66] Lieutier F, Brignolas F, Sauvard D, Yart A, Galet C, Brunet M, van de Sype H (2003). Intra- and inter-provenance variability in phloem phenols of *Picea abies* and relationship to a bark beetle-associated fungus. Tree Physiol.

[CR67] Lin Y, Yan Y (2014). Biotechnological production of plant-specific hydroxylated phenylpropanoids. Biotechnol Bioeng.

[CR68] Lindberg M, Lundgren L, Gref R, Johansson M (1992). Stilbenes and resin acids in relation to the penetration of *Heterobasidion annosum* through the bark of *Picea abies*. Eur J Forest Pathol.

[CR69] Matern U, Kneusel RE (1988). Phenolic compounds in plant disease resistance. Phytoparasitica.

[CR70] Mayr S, Siller C, Kriss M, Oberhuber W, Bauer H (2001). Photosynthesis in rust-infected adult Norway spruce in the field. New Phytol.

[CR71] Mayr S, Schwienbacher F, Beikircher B, Dämon B (2010). Damage in needle tissues after infection with *Chrysomyxa rhododendri* increases cuticular conductance of *Picea abies* in winter. Protoplasma.

[CR72] McKown AD, Klápště J, Guy RD (2014). Genome-wide association implicates numerous genes underlying ecological trait variation in natural populations of *Populus trichocarpa*. New Phytol.

[CR73] Neale DB, Savolainen O (2004). Association genetics of complex traits in conifers. Trends Plant Sci.

[CR74] Oberhuber W, Thomaser G, Mayr S, Bauer H (1999). Radial growth of Norway spruce infected by *Chrysomyxa rhododenri*. Phyton (Horn, Austria).

[CR75] Oechslin M (1933). Die *Chrysomyxa rhododendri*. Schweiz Z Forstwesen.

[CR76] Pavy N, Namroud MC, Gagnon F, Isabel N, Bousquet J (2012). The heterogeneous levels of linkage disequilibrium in white spruce genes and comparative analysis with other conifers. Heredity.

[CR77] Pavy N, Gagnon F, Rigault P (2013). Development of high-density SNP genotyping arrays for white spruce (*Picea glauca*) and transferability to subtropical and nordic congeners. Mol Ecol Res.

[CR78] Pearson TA, Manolio TA (2008). How to interpret a genome-wide association study. JAMA.

[CR79] Pezet R, Pont V (1990). Ultrastructural observations of pterostilbene fungitoxicity in dormant conidia of *Botrytis cinerea* Pers. J Phytopathol.

[CR80] Pezet R, Gindro K, Viret O, Spring JL (2004). Glycosylation and oxidative dimerization of resveratrol are respectively associated to sensitivity and resistance of grapevine cultivars to downy mildew. Physiol Mol Plant Pathol.

[CR81] Plattner K, Volgger W, Oberhuber W, Mayr S, Bauer H (1999). Dry mass production in seedlings of Norway spruce infected by the needle rust *Chrysomyxa rhododendri*. Eur J For Path.

[CR82] Porras-Hurtado L, Ruiz Y, Santos C, Phillips C, Carracedo A, Lareu MV (2013). An overview of STRUCTURE: applications, parameter settings, and supporting software. Front Genet.

[CR83] Porth I, Klápště J, McKown AD (2014). Extensive functional pleiotropy of REVOLUTA substantiated through forward genetics. Plant Physiol.

[CR84] Pré M, Atallah M, Champion A, De Vos M, Pieterse CM, Memelink J (2008). The AP2/ERF domain transcription factor ORA59 integrates jasmonic acid and ethylene signals in plant defense. Plant Physiol.

[CR85] Quesada T, Gopal V, Cumbie WP (2010). Association mapping of quantitative disease resistance in a natural population of loblolly pine (*Pinus taeda* L.). Genetics.

[CR86] Romeis T (2001). Protein kinases in the plant defence response. Curr Opin Plant Biol.

[CR87] Romsakova I, Foffova E, Kmet J, Longauer R, Pacalaj M, Gömöry D (2012). Nucleotide polymorphisms related to altitude and physiological traits in contrasting provenances of Norway spruce (*Picea abies*). Biologia.

[CR88] Schiebe C, Hammerbacher A, Birgersson JW (2012). Inducibility of chemical defences in Norway spruce bark is correlated with unsuccessful mass attacks by the spruce bark beetle. Oecologia.

[CR89] Schultz TP, Nicholas DD (2000). Naturally durable heartwood: evidence for a proposed dual defensive function of the extractives. Phytochemistry.

[CR90] Shanmugam V (2005). Role of extracytoplasmic leucine rich repeat proteins in plant defence mechanisms. Microbiol Res.

[CR91] Storey JD, Tibshirani R (2003). Statistical significance for genomewide studies. P Natl Acad Sci USA.

[CR92] Tameling WI, Vossen JH, Albrecht M (2006). Mutations in the NB-ARC domain of I-2 that impair ATP hydrolysis cause autoactivation. Plant Physiol.

[CR93] Tollefsrud MM, Kissling R, Gugerli F (2008). Genetic consequences of glacial survival and postglacial colonization in Norway spruce: combined analysis of mitochondrial DNA and fossil pollen. Mol Ecol.

[CR94] Treutter D (2006). Significance of flavonoids in plant resistance: a review. Environ Chem Lett.

[CR95] van der Beek JG, Verkerk R, Zabel P, Lindhout P (1992). Mapping strategy for resistance genes in tomato based on RFLPs between cultivars: Cf9 (resistance to *Cladosporium fulvum*) on chromosome 1. Theor Appl Genet.

[CR96] Villa-Ruano N, Pacheco-Hernandez Y, Lozoya-Gloria E, Castro-Juarez CJ, Mosso-Gonzalez C, Ramirez-Garcia SA (2015). Cytochrome P450 from plants: platforms for valuable phytopharmaceuticals. Trop J Pharm Res.

[CR97] Vogt T (2010). Phenylpropanoid biosynthesis. Mol Plant.

[CR98] Wallis C, Eyles A, Chorbadjian R, McSpadden Gardener B, Hansen R, Cipollini D, Herms DA, Bonello P (2008). Systemic induction of phloem secondary metabolism and its relationship to resistance to a canker pathogen in Austrian pine. New Phytol.

[CR99] Webb E, Broderick P, Lubbe S, Chandler I, Tomlinson I, Houlston RS (2009). A genome-wide scan of 10 000 gene-centric variants and colorectal cancer risk. Eur J Hum Genet.

[CR100] Westbrook JW, Resende MFR, Munoz P (2013). Association genetics of oleoresin flow in loblolly pine: discovering genes and predicting phenotype for improved resistance to bark beetles and bioenergy potential. New Phytol.

[CR101] Witzell J, Martín JA (2008). Phenolic metabolites in the resistance of northern forest trees to pathogens: past experiences and future prospects. Can J Forest Res.

[CR102] Xu J, Wang XY, Guo WZ (2015). The cytochrome P450 superfamily: Key players in plant development and defense. JIA.

