# Supplementary material for: Association genetics of phenolic needle compounds in Norway spruce with variable susceptibility to needle bladder rust
Source: Plant Mol Biol. 2017 Feb 11;94(3):229–51. doi: 10.1007/s11103-017-0589-5 (PMC5443855; doi:10.1007/s11103-017-0589-5)
Supplement: Supplementary file 1 — Supplementary material 1 (DOCX 23 KB) [file 11103_2017_589_MOESM1_ESM.docx]

**Supplemental Table S1.** Trees sampled for the association study. Clone code, name of the forest district, sea level (m), coordinates (Geogr. WGS1984), estimated age (years), mean *C. rhododendri* infection degree during the years 2009-2012 and classification of susceptibility are given (for details see material and methods)

| **clone** | **forest district** | **sea level** | **coordinates** | | **tree age** | **infection** | **susceptibility** |
| --- | --- | --- | --- | --- | --- | --- | --- |
| KAU-0 | Kaunertal | 1,588 | 10.743484 | 47.047103 | 30 | 3.25 | high |
| KAU-1 | Kaunertal | 1,598 | 10.743731 | 47.046697 | 30 | 2.00 | low |
| KAU-2 | Kaunertal | 1,617 | 10.743145 | 47.047455 | 30 | 2.25 | low |
| STR-0 | Strengen | 1,465 | 10.434779 | 47.123379 | 40 | 3.00 | high |
| STR-1 | Strengen | 1,667 | 10.454400 | 47.143976 | 40 | 1.00 | low |
| STR-2 | Strengen | 1,680 | 10.443636 | 47.143555 | 40 | 1.00 | low |
| STR-3 | Strengen | 1,814 | 10.446292 | 47.146386 | 50 | 1.00 | low |
| STR-4 | Strengen | 1,530 | 10.436239 | 47.121605 | 30 | 1.00 | low |
| STR-5 | Strengen | 1,745 | 10.457570 | 47.114200 | 50 | 1.00 | low |
| STR-6 | Strengen | 1,694 | 10.465687 | 47.114128 | 40 | 1.25 | low |
| FLI-0 | Fließ | 1,650 | 10.572972 | 47.112149 | 28 | 3.00 | high |
| FLI-1 | Fließ | 1,650 | 10.572972 | 47.112149 | 28 | 2.25 | low |
| LAE-0 | Längenfeld | 1,670 | 11.036697 | 47.062191 | 25 | 3.00 | high |
| LAE-1 | Längenfeld | 1,670 | 11.036697 | 47.062191 | 70 | 1.75 | low |
| LAE-2 | Längenfeld | 1,670 | 11.036697 | 47.062191 | 70 | 2.00 | low |
| LAE-3 | Längenfeld | 1,670 | 11.036697 | 47.062191 | 70 | 1.50 | low |
| HAI-0 | Haiming | 1,540 | 10.941070 | 47.228926 | 80 | 2.25 | high |
| HAI-1 | Haiming | 1,543 | 10.942484 | 47.229044 | 120 | 1.25 | low |
| SIG-0 | St. Sigmund i. S. | 1,550 | 11.136614 | 47.158348 | 80 | 2.50 | high |
| SIG-1 | St. Sigmund i. S. | 1,550 | 11.136509 | 47.158372 | 80 | 1.25 | low |
| PRA-1 | St. Sigmund i. S. | 1,578 | 11.134994 | 47.154850 | 50 | 3.50 | high |
| PRA-2 | St. Sigmund i. S. | 1,578 | 11.134917 | 47.154983 | 50 | 3.50 | high |
| PRA-3 | St. Sigmund i. S. | 1,578 | 11.135133 | 47.155010 | 50 | 3.50 | high |
| PRA-5 | St. Sigmund i. S. | 1,550 | 11.136580 | 47.158354 | 80 | 2.50 | high |
| PRA-6 | St. Sigmund i. S. | 1,503 | 11.137605 | 47.160389 | 60 | 3.25 | high |
| GRI-0 | Gries a. B. | 1,763 | 11.462670 | 47.017262 | 45 | 2.50 | high |
| GRI-1 | Gries a. B. | 1,761 | 11.462670 | 47.017262 | 50 | 1.25 | low |
| SIS-0 | Sistrans | 1,750 | 11.467312 | 47.219727 | 30 | 3.75 | high |
| SIS-1 | Sistrans | 1,650 | 11.467081 | 47.219597 | 35 | 1.50 | low |
| SIS-2 | Sistrans | 1,750 | 11.468305 | 47.219219 | 25 | 1.50 | low |
| SIS-3 | Sistrans | 1,700 | 11.466624 | 47.220002 | 25 | 1.50 | low |
| RIN-0 | Rinn | 1,650 | 11.509963 | 47.232883 | 38 | 3.75 | high |
| RIN-1 | Rinn | 1,640 | 11.515373 | 47.234699 | 38 | 1.00 | low |
| RIN-2 | Rinn | 1,680 | 11.507578 | 47.232738 | 43 | 1.25 | low |
| ALP-0 | Alpbach | 1,620 | 11.989698 | 47.374922 | 40 | 3.25 | high |
| ALP-1 | Alpbach | 1,632 | 11.976449 | 47.390044 | 30 | 1.00 | low |
| ALP-2 | Alpbach | 1,401 | 11.942708 | 47.354974 | 50 | 1.25 | low |
| ALP-3 | Alpbach | 1,625 | 11.987425 | 47.375908 | 50 | 2.25 | low |
| WIL-0 | Wildschönau | 1,483 | n.d. | n.d. | 50 | 3.50 | high |
| WIL-1 | Wildschönau | 1,435 | 12.038672 | 47.377596 | 40 | 1.25 | low |
| WIL-2 | Wildschönau | 1,517 | 12.045066 | 47.351703 | 50 | 1.75 | low |
| WIL-3 | Wildschönau | 1,539 | 12.044220 | 47.356654 | 50 | 1.75 | low |
| WIL-4 | Wildschönau | 1,440 | 12.064914 | 47.373416 | 50 | 1.75 | low |
| ELL-0 | Ellmau | 1,451 | 12.269122 | 47.493617 | 80 | 3.00 | high |
| ELL-1 | Ellmau | 1,454 | 12.269185 | 47.493535 | 35 | 1.00 | low |
| HOP-0 | Hopfgarten i.B. | 1,440 | 12.066014 | 47.322592 | 15 | 3.50 | high |
| HOP-1 | Hopfgarten i.B. | 1,440 | 12.066014 | 47.322592 | 15 | 1.00 | low |
| ASS-0 | Assling | 1,643 | 12.582846 | 46.802834 | 50 | 3.75 | high |
| ASS-1 | Assling | 1,569 | 12.581065 | 46.801860 | 50 | 1.75 | low |
| ASS-2 | Assling | 1,581 | 12.581547 | 46.802025 | 50 | 1.75 | low |
| ASS-3 | Assling | 1,593 | 12.581743 | 46.802090 | 50 | 1.75 | low |
| ASS-4 | Assling | 1,622 | 12.581941 | 46.801974 | 50 | 2.00 | low |
| ASS-5 | Assling | 1,649 | 12.582232 | 46.802749 | 50 | 2.00 | low |
| ASS-6 | Assling | 1,593 | 12.581198 | 46.800782 | 50 | 1.25 | low |
| ASS-7 | Assling | 1,600 | 12.581638 | 46.801135 | 50 | 1.75 | low |
| ASS-8 | Assling | 1,630 | 12.582898 | 46.801962 | 50 | 1.50 | low |
| ASS-9 | Assling | 1,656 | 12.583207 | 46.802324 | 50 | 1.50 | low |
| ASS-10 | Assling | 1,657 | 12.583168 | 46.802315 | 50 | 1.50 | low |
| ASS-11 | Assling | 1,643 | 12.583282 | 46.802558 | 50 | 1.50 | low |
| ASS-12 | Assling | 1,571 | 12.583101 | 46.802440 | 50 | 1.75 | low |
| ASS-13 | Assling | 1,609 | 12.583511 | 46.803037 | 50 | 2.00 | low |
| ASS-14 | Assling | 1,692 | 12.582644 | 46.803274 | 50 | 2.25 | low |
| ASS-15 | Assling | 1,738 | 12.583085 | 46.803556 | 50 | 2.25 | low |

**Supplemental Table S2.** MRM transitions and settings as well as retention time (rt) for analysed metabolites and internal standards

| **Q1 Mass (Da)** | **Q3 Mass (Da)** | **Time (msec)** | **ID** | **DP (volts)** | **CE (volts)** | **CXP (volts)** | **rt** | **Internal standard** |
| --- | --- | --- | --- | --- | --- | --- | --- | --- |
| 405.0 | 243.0 | 10 | astringin | -120 | -26 | -13 | 1.75 (trans), | orientin |
| 405.0 | 159.0 | 5 | astringin | -120 | -60 | -7 | 2.06 (cis) | orientin |
| 288.9 | 245.0 | 10 | catechin | -85 | -22 | -13 | 1.45 | orientin |
| 288.9 | 203.0 | 5 | catechin | -85 | -26 | -15 |  | orientin |
| 419.1 | 257.1 | 10 | isorhapontin | -95 | -22 | -9 | 2.10 (trans), | naringin |
| 419.1 | 241.1 | 5 | isorhapontin | -95 | -46 | -9 | 2.30 (cis) | naringin |
| 447.0 | 284.0 | 10 | kaempferol 3-glucoside | -110 | -38 | -15 | 2.22 | naringin |
| 447.0 | 255.0 | 5 | kaempferol 3-glucoside | -110 | -54 | -17 |  | naringin |
| 343.0 | 135.1 | 10 | picein-formiat | -60 | -16 | -7 | 1.19 | orientin |
| 343.0 | 44.9 | 5 | picein-formiat | -60 | -54 | -5 |  | orientin |
| 353.0 | 191.0 | 10 | chlorogenic acid | -70 | -22 | -9 | 1.48 | orientin |
| 353.0 | 85.0 | 5 | chlorogenic acid | -70 | -56 | -7 |  | orientin |
| 284.9 | 185.0 | 10 | kaempferol | -110 | -36 | -9 | 3.19 | pinosylvin |
| 284.9 | 187.0 | 5 | kaempferol | -110 | -40 | -9 |  | pinosylvin |
| 270.9 | 150.9 | 10 | naringenin | -95 | -24 | -9 | 3.09 | pinosylvin |
| 270.9 | 119.0 | 5 | naringenin | -95 | -32 | -9 |  | pinosylvin |
| 447.0 | 327.0 | 10 | orientin | -120 | -30 | -15 | 1.84 | / |
| 447.0 | 357.0 | 5 | orientin | -120 | -30 | -19 |  | / |
| 300.9 | 150.9 | 10 | quercetin | -95 | -28 | -11 | 2.81 | naringin |
| 300.9 | 178.9 | 5 | quercetin | -95 | -26 | -7 |  | naringin |
| 463.0 | 300.0 | 10 | quercetin 3-glucoside | -125 | -38 | -15 | 2.06 | naringin |
| 463.0 | 301.0 | 5 | quercetin 3-glucoside | -125 | -32 | -15 |  | naringin |
| 447.0 | 300.0 | 10 | quercitrin | -120 | -38 | -15 | 2.25 | naringin |
| 447.0 | 301.0 | 5 | quercitrin | -120 | -32 | -13 |  | naringin |
| 168.9 | 125.0 | 10 | gallic acid | -60 | -20 | -9 | 0.82 | orientin |
| 168.9 | 79.0 | 5 | gallic acid | -60 | -30 | -7 |  | orientin |
| 304.9 | 124.9 | 10 | gallocatechin | -55 | -24 | -9 | 1.06 | orientin |
| 304.9 | 179.0 | 5 | gallocatechin | -55 | -24 | -3 |  | orientin |
| 242.9 | 159.0 | 10 | piceatannol | -105 | -36 | -11 | 2.25 (trans) | naringin |
| 242.9 | 201.0 | 5 | piceatannol | -105 | -28 | -13 |  | naringin |
| 389.0 | 227.0 | 10 | piceid | -95 | -26 | -9 | 2.02 (trans), | naringin |
| 389.0 | 185.0 | 5 | piceid | -95 | -48 | -11 | 2.26 (cis) | naringin |
| 211.0 | 169.0 | 10 | pinosylvin | -90 | -26 | -5 | 3.52 | / |
| 211.0 | 167.1 | 5 | pinosylvin | -90 | -24 | -7 |  | / |
| 302.9 | 285.0 | 10 | taxifolin | -80 | -16 | -5 | 2.14 | naringin |
| 302.9 | 125.0 | 5 | taxifolin | -80 | -26 | -9 |  | naringin |
| 579.1 | 271.0 | 10 | naringin | -140 | -44 | -11 | 2.18 | / |
| 579.1 | 459.2 | 5 | naringin | -140 | -36 | -9 |  | / |
| 172.9 | 92.9 | 10 | shikimic acid | -60 | -22 | -7 | 0.37 | orientin |
| 172.9 | 111.0 | 5 | shikimic acid | -60 | -14 | -9 |  | orientin |
| 226.9 | 185.0 | 10 | resveratrol | -90 | -26 | -9 | 2.61 (trans) | naringin |
| 226.9 | 143.0 | 5 | resveratrol | -90 | -34 | -9 |  | naringin |
