# Supplementary material for: Association genetics of phenolic needle compounds in Norway spruce with variable susceptibility to needle bladder rust
Source: Plant Mol Biol. 2017 Feb 11;94(3):229–51. doi: 10.1007/s11103-017-0589-5 (PMC5443855; doi:10.1007/s11103-017-0589-5)
Supplement: Supplementary file 3 — Supplementary material 3 (DOCX 1194 KB) [file 11103_2017_589_MOESM3_ESM.docx]

| **Supplemental Table S4.** Eigenvalue, proportion of total variation explained by each principal component and cumulative proportion | | | |
| --- | --- | --- | --- |
| **PC** | **Eigenvalue** | **Proportion of total** | **Cumulative proportion** |
| PC1 | 6.582 | 0.329 | 0.329 |
| PC2 | 4.152 | 0.208 | 0.537 |
| PC3 | 2.062 | 0.103 | 0.640 |
| PC4 | 1.709 | 0.085 | 0.725 |
| PC5 | 1.527 | 0.076 | 0.802 |
| PC6 | 1.104 | 0.055 | 0.857 |
| PC7 | 0.902 | 0.045 | 0.902 |
| PC8 | 0.480 | 0.024 | 0.926 |
| PC9 | 0.431 | 0.022 | 0.947 |
| PC10 | 0.394 | 0.020 | 0.967 |
| PC11 | 0.231 | 0.012 | 0.979 |
| PC12 | 0.128 | 0.006 | 0.985 |
| PC13 | 0.109 | 0.005 | 0.991 |
| PC14 | 0.090 | 0.004 | 0.995 |
| PC15 | 0.050 | 0.003 | 0.998 |
| PC16 | 0.033 | 0.002 | 0.999 |
| PC17 | 0.016 | 7.99E-04 | 1.000 |
| PC18 | 1.69E-15 | 8.44E-17 | 1.000 |
| PC19 | 1.18E-15 | 5.92E-17 | 1.000 |
| PC20 | 4.68E-16 | 2.34E-17 | 1.000 |

| **Supplemental Table S5.** Factor loadings for the 20 phenotypic traits used to construct principal components | | | | | |
| --- | --- | --- | --- | --- | --- |
| **Trait** | **PC1** | **PC2** | **PC3** | **PC4** | **PC5** |
| *trans*-astringin | 0.325 | 0.079 | -0.239 | -0.115 | -0.037 |
| *cis*-astringin | 0.282 | -0.258 | -0.206 | -0.151 | 0.003 |
| astringin ratio | 0.056 | -0.438 | -0.022 | -0.105 | 0.041 |
| astringin sum | 0.330 | 0.050 | -0.243 | -0.122 | -0.035 |
| catechin | -0.058 | -0.047 | -0.177 | -0.096 | -0.664 |
| *trans*-isorhapontin | 0.277 | 0.077 | 0.385 | 0.233 | -0.151 |
| *cis*-isorhapontin | 0.267 | -0.108 | 0.427 | 0.136 | -0.134 |
| isorhapontin ratio | 0.149 | -0.366 | 0.248 | 0.005 | 0.024 |
| isorhapontin sum | 0.282 | 0.035 | 0.406 | 0.217 | -0.151 |
| kaempferol 3-glucoside | 0.037 | -0.152 | -0.287 | 0.587 | -0.032 |
| picein | 0.088 | -0.344 | -0.070 | -0.047 | -0.163 |
| quercetin 3-glucoside | -0.005 | 0.044 | -0.293 | 0.545 | -0.272 |
| gallocatechin | -0.035 | -0.127 | 0.034 | 0.171 | 0.367 |
| *trans*-piceatannol | 0.193 | 0.311 | -0.024 | -0.093 | 0.108 |
| *trans*-piceid | 0.340 | 0.156 | -0.143 | 0.001 | 0.019 |
| *cis*-piceid | 0.328 | -0.126 | -0.137 | -0.065 | 0.102 |
| piceid ratio | 0.093 | -0.427 | -0.042 | -0.087 | 0.113 |
| piceid sum | 0.348 | 0.124 | -0.146 | -0.008 | 0.030 |
| shikimic acid | -0.036 | 0.033 | 0.116 | -0.334 | -0.449 |
| *trans*-resveratrol | 0.218 | 0.288 | 0.041 | -0.059 | 0.127 |


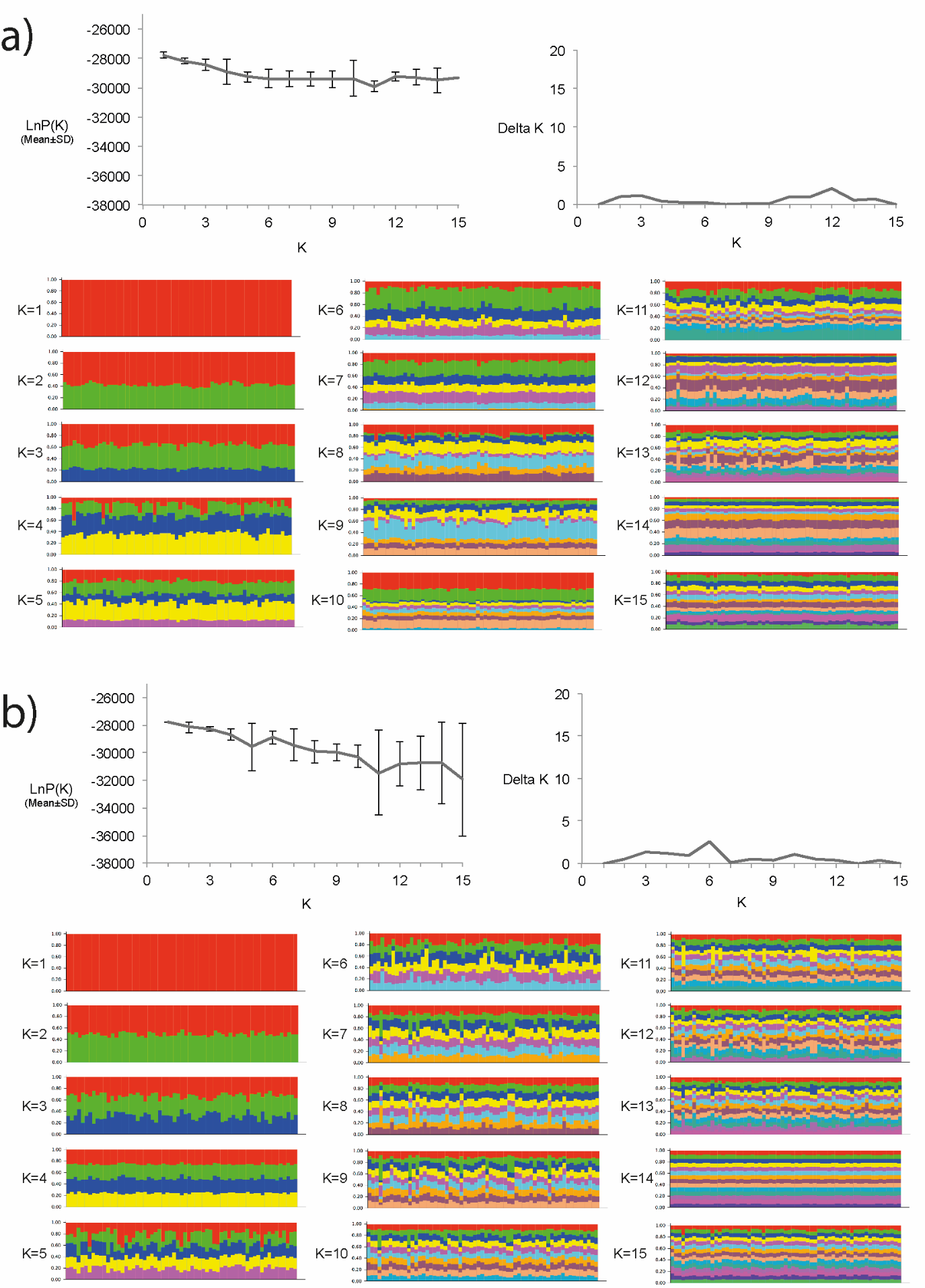


**Supplemental Figure S1. Population stratification according to STRUCTURE based on 356 SNP markers: (a) Sampling location considered (LOCPRIOR scenario), (b) sampling location not considered.** Shown are LnP (K), Delta K and bar plots. Each individual is represented by a single vertical box broken in coloured segment according to the number of assumed population K=1 to 15. Vertical box length is proportional to each cluster assignment probability (y-axis).


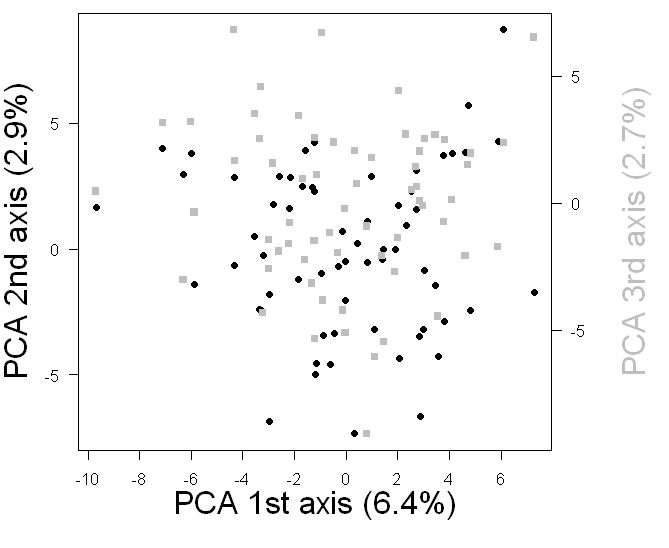


**Supplemental Figure S2. Principal component analysis of the studied population based on the genotype of 356 SNP markers.** The first three components are plotted (numbers in parenthesis refer to the proportion of explained variance, putative resistant individuals are shown in black and susceptible in grey).

(a)

(b)

(c)

**Supplemental Figure S3.** Linkage disequilibrium as a function of distance; x axis is the distance in nucleotides (bp); y axis is the correlation coefficient (r2) between SNP sites. (a) Plot obtained merging the all used SNPs from all contigs, (b) plot obtained for SNPs from contig MA_71728, (c) plot obtained for SNPs for contig MA_53529.
